# Metabolomics Analysis Identifies Sphingolipids as Key Signaling Moieties in Appressorium Morphogenesis and Function in Magnaporthe oryzae

**DOI:** 10.1128/mBio.01467-19

**Published:** 2019-08-20

**Authors:** Xiao-Hong Liu, Shuang Liang, Yun-Yun Wei, Xue-Ming Zhu, Lin Li, Ping-Ping Liu, Qing-Xia Zheng, Hui-Na Zhou, Yong Zhang, Li-Juan Mao, Caroline Mota Fernandes, Maurizio Del Poeta, Naweed I. Naqvi, Fu-Cheng Lin

**Affiliations:** a State Key Laboratory of Rice Biology, Institute of Biotechnology, Zhejiang University, Hangzhou, China; b Zhengzhou Tobacco Research Institute of CNTC, Zhengzhou, China; c Temasek Life Sciences Laboratory and Department of Biological Sciences, National University of Singapore, Singapore; d Department of Molecular Genetics and Microbiology, Stony Brook University, Stony Brook, New York, USA; e Division of Infectious Diseases, Stony Brook University, Stony Brook, New York, USA; f Veterans Affairs Medical Center, Northport, New York, USA; g Analysis Center of Agrobiology and Environmental Science, Zhejiang University, Hangzhou, China; Duke University

**Keywords:** appressorium development, early sphingolipid signaling, inducer, metabolomics analysis, pathogenicity, rice blast fungus

## Abstract

The blast fungus initiates infection using a heavily melanized, dome-shaped infection structure known as the appressorium, which forcibly ruptures the cuticle to enter the rice leaf tissue. How this process takes place remains not fully understood. Here, we used untargeted metabolomics analyses to profile the metabolome of developing appressoria and identified significant changes in six key metabolic pathways, including early sphingolipid biosynthesis. Analyses employing small molecule inhibitors, gene disruption, or genetic and chemical complementation demonstrated that ceramide compounds of the sphingolipid biosynthesis pathway are essential for normal appressorial development controlled by mitosis. In addition, ceramide was found to act upstream from the protein kinase C-mediated cell wall integrity pathway during appressorium repolarization and pathogenicity in rice blast. Further discovery of the sphingolipid biosynthesis pathway revealed that glucosylceramide (GlcCer) synthesized by ceramide is the key substance affecting the pathogenicity of Magnaporthe oryzae. Our results provide new insights into the chemical moieties involved in the infection-related signaling networks, thereby revealing a potential target for the development of novel control agents against the major disease of rice and other cereals.

## Abstract (precis)

Our untargeted analysis of metabolomics throughout the course of pathogenic development gave us an unprecedented high-resolution view of major shifts in metabolism that occur in the topmost fungal pathogen that infects rice, wheat, barley, and millet. Guided by these metabolic insights, we demonstrated their practical application by using two different small-molecule inhibitors of sphingolipid biosynthesis enzymes to successfully block the pathogenicity of M. oryzae. Our study thus defines the sphingolipid biosynthesis pathway as a key step and potential target that can be exploited for the development of antifungal agents. Furthermore, future investigations that exploit such important metabolic intermediates will further deepen our basic understanding of the molecular mechanisms underlying the establishment of fungal blast disease in important cereal crops.

## INTRODUCTION

Rice blast, caused by the ascomycete Magnaporthe oryzae, is a major challenge to crop production and global food security (1). M. oryzae utilizes appressoria to forcibly rupture the cuticle during the invasion of rice leaves (2). In addition to rice, *M*. *oryzae* also infects and causes economic damage in other cereal crops, such as wheat, barley, and millet (3). Traditional physical and chemical control methods used by cereal producers are sometimes insufficient to control rice blast outbreaks, due in part to the ability of *M. oryzae* to rapidly adapt to variation in the environment (4, 5).

Plant infection by *M. oryzae* begins with the adherence of three-celled conidia on the leaf surface, often vectored by wind or dewdrop splashes. A polarized germ tube then emerges from the spore and perceives the hard, hydrophobic surface; this process triggers appressorium formation. The initial appressoria appear as germ tube tips being swollen. It is now known that conidial nuclei are subsequently degraded by autophagy, with the content from spore cells being recycled for use by the appressoria (6). A thick layer of melanin forms on the inner side of the appressorial cell wall, and the appressoria accumulate high concentrations of compatible solute (e.g., glycerol), which generates substantial turgor pressure, thus enabling physical penetration into the host surface/tissue (7–9). During the whole development process, two rounds of mitosis are carried out in the cell. The first G_1_/S progression triggers the formation of the incipient appressorium, and a conidium nucleus migrates into the germ tube (10). The G_2_/M progression is required for appressorium maturation, when the nucleus in the germ tube undergoes mitosis to produce two daughter nuclei, of which one enters the appressorium and one returns to the conidium (11), after which the turgor pressure reaches its maximum and a new round of mitosis occurs. The second S-phase checkpoint regulates the remodeling of the septin-dependent actin cytoskeleton into toroidal networks to provide cortical rigidity and to constrain the diffusion of proteins related to pathogenesis, thereby controlling the formation of the penetration peg (11, 12). Following penetration, mitosis continues and the fungus ramifies through the host tissue.

Several signaling pathways regulate appressorium development in M. oryzae. cAMP-protein kinase A (cAMP-PKA) signaling plays an important role in surface signal recognition and appressorium morphogenesis. Inhibition of the synthesis of cAMP/PKA hinders the formation of appressoria or produces deformed appressoria. In addition, exogenous cAMP can induce appressorium formation on hydrophilic surfaces (13, 14). Three mitogen-activated protein kinase (MAPK) pathways also have been shown to regulate *M*. *oryzae* infection processes. The PMK1 MAP kinase (Pmk1 MAPK) controls appressorium formation, host penetration, and proliferation *in planta* (15), the Osm1 MAPK is dispensable for virulence but necessary for osmoregulation (16), and Mps1 MAPK is important for cell wall integrity, conidiogenesis, and host penetration (17). Additionally, target of rapamycin (TOR) signaling acts downstream from the cAMP-PKA pathway but upstream from the Pmk1 pathway and negatively controls appressorium formation by regulating the cell cycle (18, 19). Germinating conidia utilize intrinsic nutrients, such as long-chain fatty acids, to provide sufficient energy and materials for appressorial morphogenesis and for turgor generation. In the past few decades, studies on the metabolic processes occurring in appressoria have mainly focused on the transfer and utilization of carbohydrates and lipid droplets full of triacylglycerides by using gene knockout methods (20–25). Glycogen and lipid droplets are the main storage reserves present in conidia. Previous studies have revealed that lipid catabolism is a major source of glycerol needed for turgor pressure, while glycogen metabolism is involved in the energy supply during the infection process (23, 24). Nevertheless, relatively little is known about other metabolic pathways that are active in developing appressoria. To date, there have been studies that characterized changes in the transcriptomes and proteomes throughout the course of appressorial development (26, 27), but we are unaware of an analysis of the metabolome in *M. oryzae*. As it presents cellular information that is downstream from genomics, transcriptomics, and proteomics data, metabolomics is widely held to represent the most representative phenotype of an organism in a given context (28).

In this study, untargeted metabolomics analysis during various stages of appressorial development identified significant changes in six key metabolic pathways. Several trends in differentially accumulated metabolites suggested interesting biological insights related to energy and sterol metabolism. Specifically, our metabolomics data suggested that early sphingolipids are essential for the development of appressoria in *M. oryzae*. We investigated the role(s) of sphingolipid biosynthesis in the pathogenicity of *M. oryzae* using small-molecule-inhibitor- and genetic-knockout-based interruption of enzymatic function, as well as both genetic and chemical complementation experiments. Collectively, these experiments revealed that the sphingolipid biosynthesis pathway is essential for normal appressorial development and pathogenicity of *M*. *oryzae*. Our study further demonstrated that the abnormal morphology of appressoria caused by lack of ceramides is related to cell cycle regulation and that ceramide acts upstream from protein kinase C signaling, which is essential for pathogenicity, thereby demonstrating that early sphingolipids are indispensable for the initiation of the devastating blast disease in rice.

## RESULTS

### Profiling of the appressorial metabolome.

**FIG 1:**
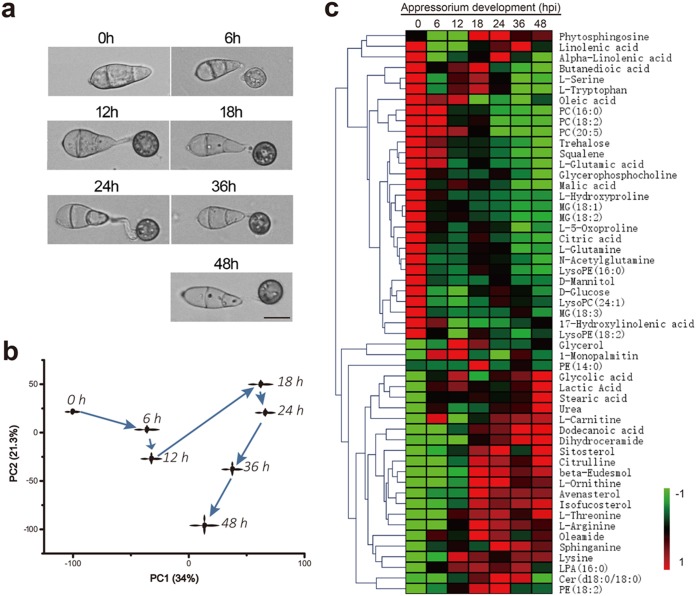
Metabolome profiling during appressorial development in *M. oryzae*. (a) Micrographs of appressoria at different time points (scale bar = 10 μm). (b) PCA score scatter plot depicting time-dependent trajectory of metabolic profiles during appressorium development. Each dot represents the averaged metabolic status of six biological replicates. Two principal components (PC1 and PC2) described 55.3% of the total variance at logarithmic scale, and the transformation of the seven time point dots mainly changed in the direction of the first principal component (PC1); the difference continued to grow between 0 and 24 h and then decreased from 24 to 48 h. (c) Relative levels of differential metabolites during appressorium formation. Data have been normalized to unit variance and are mean centered (with six replicates). Comparisons were generated via hierarchical cluster analysis (HCA) using an average linkage method based on Euclidian distance. Shades from green to red represent the increasing metabolite levels in an appressorium. PC, phosphatidylcholine; PE, phosphatidylethanolamine; MG, monoglyceride; LPA, lysophosphatidic acid.

Conidia were germinated on a hydrophobic surface and harvested at seven time points (0 h, 6 h, 12 h, 18 h, 24 h, 36 h, and 48 h) of appressorial development that were established in previous functional genomics studies (27, 29, 30). Samples at hour zero were ungerminated spores, incipient appressoria form during the initial 6 to 12 h, and the period from 12 to 24 h represents the mature appressoria. In seeking to better understand the mature appressorium, we extended the incubation time to 48 h (Fig. 1a). Gas chromatography-mass spectrometry (GC-MS) and liquid chromatography (LC)-MS analyses yielded a total of 10,344 ion signals (9,631 by LC-MS and 713 by GC-MS). These untargeted metabolomics data were initially explored via principal-component analysis (PCA), which revealed that the 42 samples (6 replicates for each of the 7 time points) were clustered into seven groups that were well separated, highlighting notable transformations of metabolic phenotypes at the different time points. A total of 110 metabolites were structurally identified from the GC-MS data based on spectral matching against a reference database. Tandem MS (MS/MS) analysis of LC-MS candidate metabolites enabled the structural identification of 29 metabolites via spectral comparison and matching with reference standards (Table S1a and c in the supplemental material).

10.1128/mBio.01467-19.10 TABLE S1 (a) List of metabolites identified in appressoria by GC-MS. (b) List of 774 ion signals detected by LC-MS that have different abundances at 0 h and 24 h in appressoria. (c) List of metabolites that have different abundances at 0 h and 24 h in appressoria. (d) Significant difference analysis of appressorium morphotypes under different concentrations of inhibitors. (e) Genes involved in ceramide synthesis in *M. oryzae*. (f) Conidiation of Δ*Molag1* and Δ*Molag1/Molag1* strains. (g) Primers used in this study. (h) Significant difference analysis for appressoria that underwent cytorrhysis. (i) Significant difference analysis of appressorium morphotypes under inhibitors. (j) Conidiation of Δ*Mocgt1* and Δ*Mocgt1/Mocgt1* strains. Download Table S1, XLSX file, 0.1 MB. Copyright © 2019 Liu et al. 2019 Liu et al. This content is distributed under the terms of the Creative Commons Attribution 4.0 International license.

**FIG 2:**
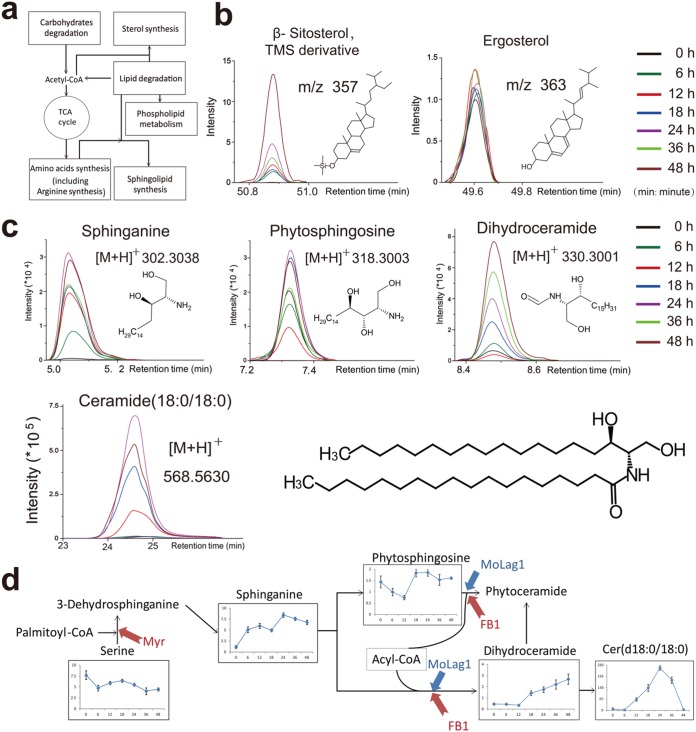
Pathway enrichment analysis in appressoria. (a) Schematic map of pathways with significant enrichment in appressoria. TCA, tricarboxylic acid. (b) Sitosterol but not ergosterol accumulates in appressoria. Overlaid ion chromatograms (*m/z* = 172 and *m/z* = 363) of GC-MS-analyzed extract samples from different time points after sample derivatization by alkylation. The molecular structures of the two metabolites represent the alkylated forms of the metabolites. TMS, trimethylsilyl. (c) Sphingolipids accumulate during pathogenic development. Overlaid ion chromatograms of LC-MS-analyzed samples showing MS analysis of sphingolipids from appressoria. Chemical structure of a ceramide (d18:0/18:0) is shown. (d) Early sphingolipid synthesis in appressoria of *M. oryzae*. Graphical representation of the abundance of metabolites related to sphingolipid metabolism over the time course. Data represent the mean values ± standard deviations (SD) from six replicates. Red arrows indicate the corresponding steps of the pathway inhibited by myriocin (Myr) or FB1, and blue arrows indicate the biosynthetic steps catalyzed by MoLag1.

A time-dependent trajectory analysis of the metabolic profiles for different stages of pathogenic development indicated that the metabolic profile of appressorial samples at 24 h, a key point of the infection process for the blast fungus, was least similar to that of the ungerminated conidia (Fig. 1b). Orthogonal projection to latent structures discriminant analysis (OPLS-DA) between the 0-h and 24-h samples was carried out for both the GC-MS and LC-MS data (Fig. S1c to f), and a combination of variable importance in projection (VIP) value analysis and Student’s *t* test revealed 29 differentially accumulated metabolites in the GC-MS data set and 774 differentially accumulated ions (with 29 identified structurally) in the LC-MS data set (Fig. 1c; Table S1b and c). We next assigned such differentially accumulated metabolites to biosynthesis pathways based on the Kyoto Encyclopedia of Genes and Genomes (KEGG) database and also manually determined whether any of them have been reported as occurring in previously studied M. oryzae metabolic processes. The KEGG analysis that compared the 0-h and 24-h samples revealed significant enrichment for six pathways: degradation of lipids, degradation of carbohydrates, arginine synthesis, sphingolipid synthesis, sterol synthesis, and phospholipid metabolism (Fig. 2a; Fig. S2 and S3).

10.1128/mBio.01467-19.1 FIG S1 Data visualization analysis. (a and b) Comparison of methods for collecting materials. In the two groups of PCA analysis, data from 0 h are significantly separated from data from 24 h, and the data points represent aggregates. Method 2 results (b) are more compact than method 1 results (a). From the summary of principal components, the first principal component of method 1 (a) accounts for 44.4% of the total data, and the first four principal components together account for 83.6% of the data. The first principal component of method 2 (b) accounts for 77.2% of the total data, and the first two components can account for 90% of all data. (c and e) OPLS-DA score plots separating the results for 0-h and 24-h samples that were GC-MS analyzed/LC-MS analyzed, respectively. (d and f) V plots indicating significantly altered metabolites/ions identified in GC-MS/LC-MS data sets, respectively. Download FIG S1, TIF file, 1.4 MB. Copyright © 2019 Liu et al. 2019 Liu et al. This content is distributed under the terms of the Creative Commons Attribution 4.0 International license.

10.1128/mBio.01467-19.2 FIG S2 Proposed model of metabolic coregulation between carbohydrate and fatty acid degradation in appressorium of *M*. *oryzae*. Diagrams indicate the abundances of metabolites over the time course. Data represent the mean values ± standard deviations (SD) for six replicates. The pathway map refers to related research results in the rice blast fungus (10–16). Download FIG S2, TIF file, 1.7 MB. Copyright © 2019 Liu et al. 2019 Liu et al. This content is distributed under the terms of the Creative Commons Attribution 4.0 International license.

10.1128/mBio.01467-19.3 FIG S3 Metabolism of sitosterol, arginine, and phospholipid in appressoria of *M*. *oryzae*. (a and b) Biosynthesis of sitosterol and arginine in appressorium. Diagrams show the abundances of metabolites during the time course. Data represent the mean values ± SD for six replicates. (c) Schematic diagrams of phospholipid metabolism. Diagrams show the abundances of metabolites during the time course. Data represent the mean values ± SD for six replicates. (d) Changes in the contents of phospholipids detected during the time course of incubation. Data have been normalized to unit variance and mean centered. Shades from green to red represent the increasing metabolite levels in appressoria. Download FIG S3, TIF file, 1.4 MB. Copyright © 2019 Liu et al. 2019 Liu et al. This content is distributed under the terms of the Creative Commons Attribution 4.0 International license.

### Significant changes in energy, sterol, and sphingolipid metabolism during appressorial development.

The identities of the differentially accumulated metabolites provided biological insights about appressorial development and function. For example, while we detected the accumulation of sitosterol and its precursor metabolites (e.g., avenasterol and isofusterol), we did not find significant changes in the levels of fungal sterols (ergosterol) at the aforementioned 7 time points during appressorium formation (Fig. 2b). This was deemed to be an interesting finding in light of recent research that showed that an exogenous application of phytosterols induces α-1,3-glucan accumulation in M. oryzae cell walls and facilitates colonization of host tissues (31). We also found that l-lactic acid accumulation occurred at the same time as lipid degradation (e.g., free fatty acid and glycerol arising from the breakdown of monoacylglycerol) (Fig. S4). This observation suggests the possibility that the known redox imbalance caused by lipid catabolism in peroxisomes may be counterbalanced by the increased levels of NAD^+^ that result from lactic acid biosynthesis (22, 23).

10.1128/mBio.01467-19.4 FIG S4 l-Lactic acid accumulation coincides with lipid metabolism. (a) l-Lactic acid accumulates in appressoria in the blast fungus. Overlaid ion chromatograms (*m/z* = 117) of GC-MS-analyzed extract samples from different time points after sample derivatization by alkylation. (b) Continuous degradation of triacylglycerol mediated by glycerolysis. The final product of lipid droplet degradation is glycerol, and fatty acid chains of different lengths are decomposed during the degradation process. (c) Changes in the contents of metabolites involved in glycerolysis detected during the time course of incubation. Data have been normalized to unit variance and mean centered. Shades from green to red represent the increasing metabolite levels in appressoria. Download FIG S4, TIF file, 1.8 MB. Copyright © 2019 Liu et al. 2019 Liu et al. This content is distributed under the terms of the Creative Commons Attribution 4.0 International license.

We were most intrigued by trends in the *de novo* synthesis of sphingolipids. Specifically, we found that early intermediates of the sphingolipid biosynthesis pathway (sphinganine, phytosphingosine, dihydroceramide, and ceramide) were accumulated during appressorium formation (Fig. 2c). The amounts of ceramide were extremely low in ungerminated conidia (0 h) and in incipient appressoria (6 h). During appressorium maturation (12 h, 18 h, and 24 h), ceramide accumulated drastically. After reaching a peak value at 24 h, it decreased promptly with the increase of time (24 h, 36 h, and 48 h). By 48 h, the ceramide level was undetectable (Fig. 2d). We conclude that metabolites linked to the ceramide biosynthesis pathway accumulate specifically during appressorial morphogenesis and are metabolized during the maturation stage.

### Chemical inhibition of early sphingolipid biosynthesis.

**FIG 3:**
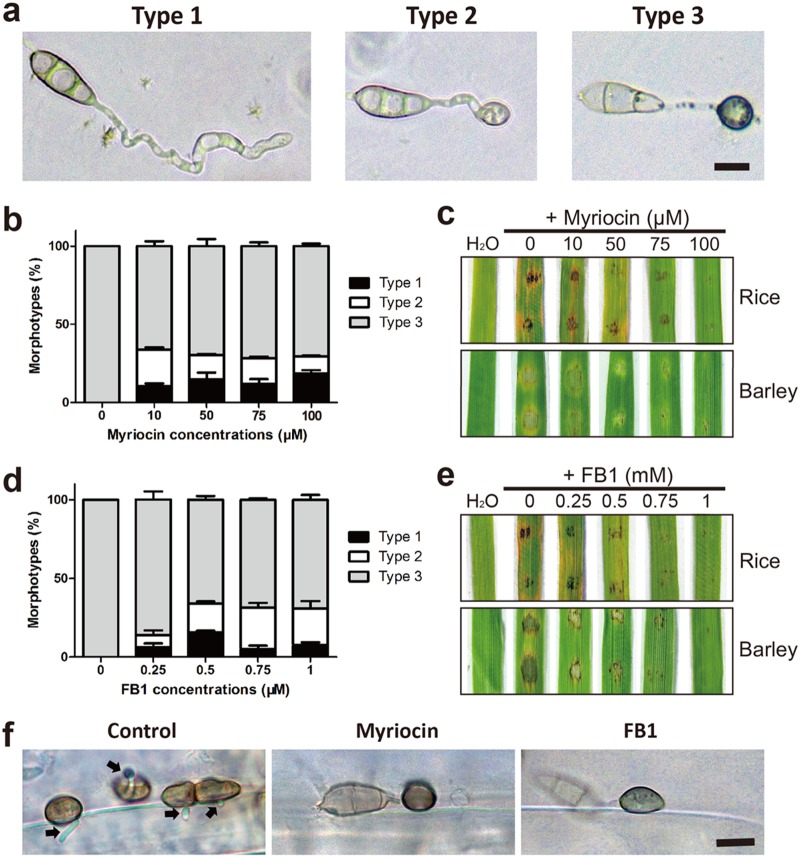
Chemical inhibition of ceramide biosynthesis impairs appressorium formation and pathogenicity. (a) Appressorial development with different maturity states caused by myriocin/FB1 inhibitors (scale bar = 10 μm). (b and d) Proportions of the three phenotypes at different concentrations of myriocin and FB1. Data represent the mean values ± SD (*n* = 100, three independent experiments). The results of inferential statistical analyses for each concentration are presented in Table S1d in the supplemental material. (c and e) Attenuated virulence of strain Guy11 (wild type) upon exposure of germinating conidia to various concentrations of myriocin/FB1. Conidial suspensions were inoculated onto rice and barley leaves to allow disease development. Disease symptoms were photographed at 4 days postinoculation (dpi). (f) Ceramide synthesis inhibitors prevent the formation of penetration pegs in appressoria. Infected barley leaf cells at 24 hpi without or with myriocin (100 μM)/FB1 (1 mM) were observed by light microscopy following the decolorisation of leaf tissue (scale bar = 10 μm). Penetration pegs are marked with arrows.

We sought to further investigate the role of early intermediates of the sphingolipid pathways in M. oryzae, since very little is known about the role of such lipid signaling in general in fungi. We utilized myriocin (32) to chemically inhibit the serine palmitoyltransferase, the key enzyme in sphingosine biosynthesis. Treatment of wild-type *M. oryzae* (strain Guy11) with various concentrations of myriocin had no effect on conidial germination. However, such myriocin treatment seriously affected the morphology of appressoria, resulting in three morphotypes with distinctly different maturity states (Fig. 3a and b). Morphotype 1 was characterized by irregular swelling at the germ tube tip with no appressoria, morphotype 2 possessed incipient appressoria with a thin layer of melanin, and morphotype 3 was normal appressoria indistinguishable from those of wild-type *M. oryzae*. Likewise, treatment with fumonisin B1 (FB1), an inhibitor of sphingosine *N*-acyltransferase (32), also resulted in three highly similar defects/morphotypes (Fig. 3d; Table S1d).

Next, we conducted a detailed analysis of the effects of these ceramide synthesis inhibitors on the pathogenicity of M. oryzae in assays using the detached leaves of barley and rice. Both myriocin and FB1 acted in a dose-dependent manner to prevent pathogenicity of the rice blast fungus (Fig. 3c and e). While leaves inoculated with untreated *M. oryzae* conidial suspensions showed typical blast disease symptoms, there were almost no necrotic lesions on leaves inoculated with inhibitor-treated conidia (100 μM myriocin or 1 mM FB1). Furthermore, live-cell imaging at 48 h postinoculation (hpi) showed that treatment with 100 μM myriocin rendered appressoria incapable of penetrating the host surface (Fig. S5a and b). To elucidate the defect in appressorium penetration caused by myriocin/FB1, we measured the internal turgor pressure within appressoria using an incipient cytorrhysis assay (9). However, no significant difference was observed between control and inhibitor-treated appressoria (Fig. S5c; Table S1h). We further observed the ability of penetration pegs to form: live-cell imaging at 24 hpi showed that treatment with inhibitors prevented the formation of penetration pegs of appressoria in *M. oryzae* (Fig. 3f). Thus, we conclude that ceramide synthesis inhibitors affect appressorium morphology, as well as penetration peg formation, which accounts for the failure of appressoria to infect host cells upon exposure to these chemicals.

10.1128/mBio.01467-19.5 FIG S5 Chemical inhibition and complementation. (a) Prevention of penetration of appressoria in epidermal cells of barley leaves by myriocin application. Infected epidermal cells at 48 hpi without or with myriocin (100 μM) were decolorized and analyzed by light microscopy (scale bar = 20 μm). (b) Percentages of appressorial penetration without or with myriocin (100 μM). Data represent the mean values ± SD (*n* = 100, three independent experiments). Data were analyzed with two-tailed *t* test comparison. Values marked with asterisks are statistically significant. **, *P* < 0.001 (df = 4, *P* = 0.000). (c) Percentages of appressoria undergoing cytorrhysis under different concentrations of glycerol. Data represent the mean values ± SD (*n* = 100, three independent experiments). Significant difference analyses of data are given in Table S1h. (d) Addition of exogenous ceramide restored the ability of the Δ*Molag1* mutant to penetrate barley leaf cells. Infected leaves at 48 hpi without or with C_2_-ceramide (100 μM) were decolorized prior to microscopic observation of the infected cells in the epidermis (scale bar = 20 μm). Download FIG S5, TIF file, 2.5 MB. Copyright © 2019 Liu et al. 2019 Liu et al. This content is distributed under the terms of the Creative Commons Attribution 4.0 International license.

### Gene deletion analysis and genetic/chemical complementation of early sphingolipid biosynthesis.

We next sought genetic evidence to corroborate our chemical inhibitor studies on the role of ceramides in *Magnaporthe* pathogenicity. Yeast (Saccharomyces cerevisiae) has 6 genes known to encode enzymes that function in *de novo* synthesis of ceramide (33). We identified the orthologs for six of these genes in the *M*. *oryzae* genome (Table S1e). We used the targeted gene replacement strategy to generate gene knockout mutants for each of these six genes. However, we were only able to obtain a null mutant for *M*. *oryzae LAG1* (*MoLAG1*; MGG_03090), which functions in the synthesis of ceramide from acyl-coenzyme A (CoA) and dihydrosphingosine or phytosphingosine. It is notable that the other 5 genes function at the preceding steps of the early sphingolipid biosynthesis pathway, thus suggesting that early sphingolipid metabolites are essential for cell viability in *M. oryzae*.

**FIG 4:**
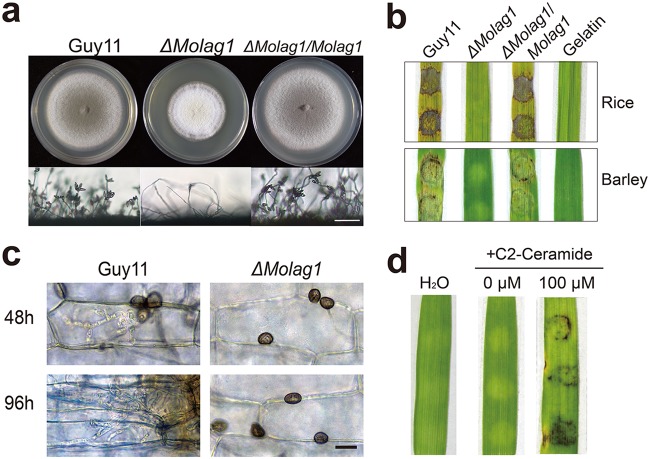
Biological functions of *MoLAG1*. (a) Colony growth and conidiophores of the indicated *M*. *oryzae* strains (scale bar = 50 μm). The Δ*Molag1* mutant showed reduced mycelial growth and dramatically reduced sporulation. Data from inferential statistical tests of the conidiation assay results are presented in Table S1f. (b) Loss of virulence of the Δ*Molag1* mutant. Mycelial discs were inoculated onto rice and barley leaves to allow disease development. Disease symptoms were photographed at 4 dpi. (c) Live-cell imaging at 48 hpi and 96 hpi of Guy11 and Δ*Molag1* strains inoculated onto barley leaves showed that the Δ*Molag1* mutant cannot penetrate the host cuticle (scale bar = 20 μm). (d) Application of exogenous ceramide restored pathogenicity in the Δ*Molag1* mutant. Mycelium discs were inoculated onto barley leaves to allow disease development. Blast disease symptoms were photographed at 4 dpi.

In yeast, *LAG1* encodes sphingosine *N*-acyltransferase, an essential subunit of the acyl-CoA-dependent ceramide synthase enzyme complex (33). Prior to investigating the role of *MoLAG1* in pathogenicity, we examined the growth of a Δ*Molag1* mutant and found that, compared to the growth of wild-type M. oryzae (Guy11), the Δ*Molag1* mutant showed impaired mycelial growth in colony assays and exhibited a severe reduction in the extent of conidiation or asexual reproduction (Fig. 4a; Table S1f). To assess pathogenicity, mycelial plugs of the Δ*Molag1* mutant were inoculated onto detached rice and barley leaves. No blast disease symptoms were found on Δ*Molag1* mutant-inoculated leaves, while the wild-type Guy11 generated extensive necrotic lesions (Fig. 4b). Moreover, microscopic observation of inoculated leaf tissues showed that the appressorium-like structures of the Δ*Molag1* mutant are unable to penetrate the host surface, even after 96 hpi (Fig. 4c).

Genetic complementation revealed that the reintroduction of the *MoLAG1* gene into the Δ*Molag1* mutant restored all of its normal phenotypes. That is, conidia of the Δ*Molag1* mutant complemented with *MoLAG1* grew normally, exhibited proper conidiation, and could penetrate barley or rice leaves, as well as generating necrotic lesions (Fig. 4a to c). Furthermore, we conducted chemical complementation experiments in which we supplemented Δ*Molag1* strain mycelia with exogenous ceramide (in growth medium) to further verify that the interruption of the acylation of sphingoid bases in early sphingolipid biosynthesis caused by knockout of *MoLAG1* was indeed responsible for the observed pathogenesis defects. Hyphae of the Δ*Molag1* mutant grown in liquid CM for 72 h were inoculated onto the surface of the barley leaves for pathogenesis analysis. While untreated Δ*Molag1* strain hyphae did not cause necrotic lesions on barley leaves, obvious lesions were apparent on leaves inoculated with hyphae that were treated with 100 μm C_2_-ceramide (Fig. 4d; Fig. S5d). Thus, genetic and chemical complementation were both able to restore the pathogenicity of the Δ*Molag1* mutant. We conclude that MoLag1/ceramide biosynthesis is essential for proper pathogenic growth and development in the rice blast fungus.

### Inhibition of ceramide biosynthesis impairs the cell cycle in *M. oryzae*.

**FIG 5:**
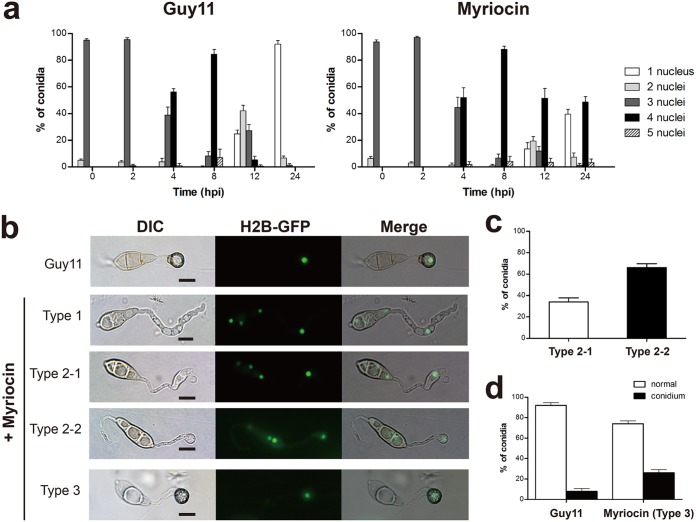
Inhibition of ceramide biosynthesis results in disorder of the cell cycle. Guy11 conidia were inoculated onto artificial hydrophobic surfaces. (a) Numbers of nuclei carried by germinating Guy11 H2B:GFP conidia without or with myriocin (100 μM) at the indicated time points. Data represent the mean values ± SD (*n* = 100, three independent experiments). (b) At 24 hpi, the wild type only has one nucleus inside the appressorium (scale bar = 10 μm). For morphotypes induced by myriocin, spore germination of type 1 and type 2-1 showed three nuclei in the conidium and one nucleus in the germ tube; conidia of type 2-2 showed three nuclei in the conidium and one nucleus in the appressorium; and in type 3, nuclei migrated successfully into the appressorium, similarly to nuclei in the wild type. (c) Proportions of the two nuclear migration states in the second morphotype at 24 hpi. Data represent the mean values ± SD (*n* = 100, three independent experiments). (d) Proportions of nuclear degradation in the wild type and morphotype 3 caused by myriocin at 24 hpi. Data represent the mean values ± SD (*n* = 100, three independent experiments).

We hypothesize that the different degrees of maturation of the appressorium caused by inhibitors are related to cell cycle regulation, and Guy11 conidia expressing histone H2B fused to green fluorescent protein (GFP) (Guy11 H2B:GFP) without or with myriocin (100 μM) were inoculated for observation. In the early 8-hpi time point, the inhibitor had no significant effect on the number of nuclei, while during the time from 12 to 24 hpi, when the number of nuclei in the wild-type strain Guy11 gradually degraded and only one nucleus remained in each appressorium, the addition of inhibitors affected the degradation of nuclei (Fig. 5a). Further observations in samples from the early 8-hpi time point showed that Guy11 H2B:GFP carried three nuclei in each conidium and one in each appressorium, while in the presence of inhibitors, some cells had three nuclei in each conidium but one nucleus per germ tube and none in the appressoria. By 24 hpi, the nucleus in the germ tube still failed to enter the appressorium, and it subsequently degraded in the conidium (Fig. 5b, type 1 and type 2-1). Some nuclei could successfully enter the appressoria, but the nuclei in the conidia were not degraded (Fig. 5b, type 2-2). The majority of type 3 cells (∼70%) completed conidial nuclear degradation and carried a single appressorial nucleus, similarly to the Guy11 H2B:GFP conidia (Fig. 5b to d). The occurrence of new nuclei clearly indicates that the inhibition of early sphingolipids does not affect the interphase (G_1_, S, and G_2_ phase) and metaphase of mitosis, but the failure of nuclei to enter into the appressoria still indicates that the absence of ceramide impaired the cell cycle in *M. oryzae*. We reason that ceramides are essential for normal appressorial development by controlling the late stages of cell cycle progression.

Moreover, the transfer of glycogen and lipid droplets from conidia to appressoria was restricted by ceramide synthesis inhibitors in all three of the morphotypes (Fig. S6). We infer that chemical inhibition of sphingolipid biosynthesis disrupts proper appressorium development via several mechanisms, including cell cycle disorder, deregulation of nuclear degradation, and disruption of bulk biochemical and/or metabolism-signaling-specific intermediates.

10.1128/mBio.01467-19.6 FIG S6 Chemical inhibition of ceramide biosynthesis impairs utilization of nutrient reserves in appressoria. (a) Cellular distribution of glycogen during appressorium development. Conidial suspensions without or with myriocin (100 μM) were incubated on hydrophobic plastic coverslips for appressorium formation. Sample coverslips were removed every 8 h and incubated in KI/I2 for observation. Yellowish-brown glycogen deposits are visible after 1 min of incubation. Glycogen is present in conidia at 0 h and is almost completely transferred to appressoria by 8 h; glycogen in appressoria is then hydrolyzed and utilized. By 24 hpi, the glycogen in appressoria of the wild type is fully consumed, whereas the transfer of glycogen to appressoria was severely hindered for all morphotypes after treatment of germinating conidia with myriocin (scale bar = 10 μm). (b) Cellular distribution of lipid droplets during appressorium morphogenesis. With the same experimental setting as described for panel a, germinating conidia were visualized via Nile red staining. Lipid droplets in red (right) and fungal structures in bright-field view (left) are displayed. Lipid droplets are present in conidia at 0 h, after which they are degraded. After 24 hpi, lipid droplets in appressoria were fully degraded in the wild type, while myriocin treatment severely hindered the degradation of lipid droplets (scale bar = 10 μm). (c) Proportions of glycogen and lipid droplet degradation in wild type and morphotypes 1 to 3 caused by myriocin at 24 hpi. Data represent the mean values ± SD (*n* = 100, three independent experiments). Download FIG S6, JPG file, 1.3 MB. Copyright © 2019 Liu et al. 2019 Liu et al. This content is distributed under the terms of the Creative Commons Attribution 4.0 International license.

### Ceramide regulates the PKC-CWI signaling pathway in *M. oryzae*.

**FIG 6:**
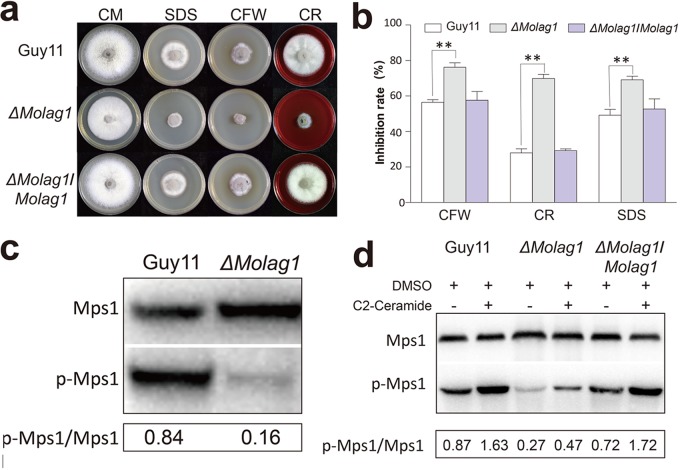
Defects of Δ*Molag1* mutant in response to CWI pathway. (a) The wild type (Guy11), Δ*Molag1* mutant, or Δ*Molag1/MoLAG1* mutant was cultured for 8 days on complete medium (CM) with or without 200 μg/ml calcofluor white (CFW), 200 μg/ml Congo red (CR), or 0.01% (vol/vol) SDS. (b) The Δ*Molag1* mutant showed strong sensitivity to cell wall stress. Data represent the mean values ± SD (with six replicates) and were analyzed using one-way ANOVA followed by *post hoc* Tukey’s honestly significant difference (HSD) test for multiple pairwise comparisons. Values marked with asterisks are statistically significant. **, *P* < 0.001. (c) Phosphorylation of Mps1 in Guy11 and the Δ*Molag1* mutant. Total protein was isolated from mycelia grown in liquid CM for 72 h. Three independent replicates were compared and showed similar results. (d) Phosphorylation of Mps1 in Guy11, Δ*Molag1*, and Δ*Molag1/MoLAG1* strains. Total protein was isolated from mycelia grown in liquid CM with or without 100 μM C_2_-ceramide for 72 h. Three independent replicates were compared and showed similar results. DMSO, dimethyl sulfoxide.

The developmental defects in appressorium formation caused by ceramide synthesis inhibitors indicated that early sphingolipid biosynthesis is likely involved in the infection-related signaling pathways in *M. oryzae*. Three major signaling cascades have been implicated in appressorium morphogenesis: cAMP-PKA, TOR, and the protein kinase C-mediated cell wall integrity (PKC-CWI) pathway (4, 34). We therefore explored the relationship between ceramide and these three signaling pathways. It has been proved that appressorium formation can be remediated by cAMP treatment in PKA-deficient mutants or by treatment with the specific TOR kinase inhibitor rapamycin in a TOR-deficient mutant (18). Our investigation found that neither exogenous cAMP nor rapamycin could suppress the phenotypic defects caused by myriocin treatment, demonstrating that ceramide signaling does not act upstream from cAMP-PKA and the TOR pathway (Fig. S7a; Table S1i). However, the Δ*Molag1* mutant showed strong sensitivity to cell wall stressors, including sodium dodecyl sulfate (SDS), calcofluor white (CFW), and Congo red (CR), indicating a potential role for ceramide in the PKC-CWI pathway (Fig. 6a and b). PKC inhibition via chelerythrine chloride or Go 6983 (35) was shown to result in appressorial morphotypes similar to those caused by myriocin or FB1 treatment (Fig. S7b). In the rice blast fungus, Mps1 functions downstream from PKC for appressorium repolarization (4, 34). We found that the loss of ceramide signaling (Δ*Molag1*) led to significantly reduced phosphorylation of Mps1 (Fig. 6c) and that exogenously added ceramide partially restores such Mps1 phosphorylation in the Δ*Molag1* mutant (Fig. 6d). We conclude that ceramide signaling modulates the PKC-based cell wall integrity pathway, in addition to its important role in regulating cell cycle-dependent appressorial morphogenesis and function in the rice blast pathogen.

10.1128/mBio.01467-19.7 FIG S7 Ceramide acts upstream from the PKC-CWI signaling pathway instead of the PKA or TOR signaling. (a) Neither cAMP (10 mM) nor rapamycin (100 nM) treatment restored appressorium morphotypes caused by myriocin. Proportions of the three phenotypes were scored at 24 hpi. Data represent the mean values ± SD (*n* = 100, three independent experiments). Significant difference analysis of appressorium morphotypes are given in Table S1i. (b) PKC inhibitors result in phenotypes of appressoria similar to those caused by myriocin or FB1. The concentrations of the inhibitors were 10 μM for chelerythrine chloride and 100 μM for Go 6983. The proportions of the three morphotypes were scored at 24 h. Data represent the mean values ± SD (*n* = 100, three independent experiments). Download FIG S7, TIF file, 1.0 MB. Copyright © 2019 Liu et al. 2019 Liu et al. This content is distributed under the terms of the Creative Commons Attribution 4.0 International license.

### GlcCers synthesized from ceramides are responsible for the pathogenicity of *M. oryzae*.

**FIG 7:**
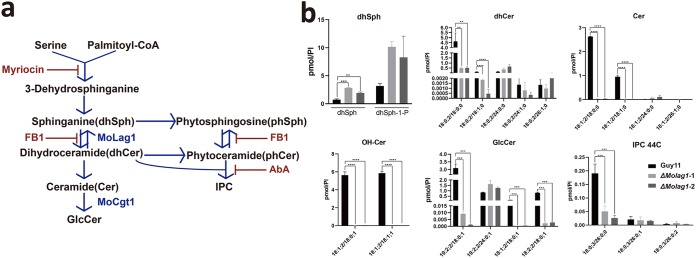
MoLag1 mainly synthesizes ceramides for glucosylceramides. (a) Sphingolipid metabolic pathways in fungi. Red indicates the corresponding steps of the pathways inhibited by myriocin, FB1, and AbA. Blue indicates the biosynthetic steps catalyzed by MoLag1 and MoCgt1. (b) The Δ*Molag1* mutant showed a reduced abundance of GlcCer precursors (dhCer, OH-Cer, and Cer) and, ultimately, reduced production of GlcCers. Guy11 and two *MoLAG1* knockout transformants were used for mass spectrometry to detect sphingolipids. Each lipid amount (in pmol) was normalized by the abundance of PI (pmol/PI). dh, dihydro; IPC 44C, IPCs of 44 carbons. **, *P* < 0.01, ***, *P* < 0.001, and ****, *P* < 0.0001 when groups were compared using one-way ANOVA with Dunnet’s posttest. See panel A for abbreviations.

Thus far, we can conclude that by regulating the cell cycle and PKC signaling, early sphingolipids (ceramides) affect the pathogenicity of *M. oryzae* (see Fig. 9). However, ceramides are the precursors of glucosylceramide (GlcCer) and inositol phosphorylceramide (IPC), which are the end products of late sphingolipid biosynthesis (Fig. 7a). Is the loss of pathogenicity in the Δ*Molag1* mutant, then, attributable to the lack of GlcCer or IPC or of both? To solve this problem, phylogenetic analysis and sequence comparison of the Lag1 motif (36) were performed on the ceramide synthases of six fungi, and the results showed that MoLag1 had the highest homology with Bar1 of Fusarium graminearum (FgBar1), which is involved in the production of GlcCers in F. graminearum (Fig. S8a and b) (37). We therefore inferred that MoLag1 is likely responsible for the synthesis of GlcCers.

10.1128/mBio.01467-19.8 FIG S8 MoLag1 mainly synthesizes ceramides for GlcCers. (a) Phylogenic tree of Lag1 protein homologs. The sequences of ceramide synthase genes were obtained from NCBI. The tree was built by comparing the deduced amino acid sequences of members of the Lag1 protein family from Magnaporthe oryzae, Saccharomyces cerevisiae, Schizosaccharomyces cerevisiae, Candida albicans, Fusarium graminearum, and Aspergillus nidulans. ScLag1, ScLac1, CaLag1, SpLac1, AnLagA, FgLagA, and MoLac1 are one group, while SpLag1, CaLac1, AnBarA, FgBar1, and MoLag1 are another group. The tree was constructed based on the principle of maximum-likelihood estimation, with bootstrap support values higher than 70% above the branches. InL (Log likelihood) = −7,129.73. (b) Alignment of the Lag1 motif in the Lag1 homologs. (c) Sphingolipid levels in Guy11 and Δ*Molag1* strains. Guy11 strain and two MoLag1 knockout transformants were used for mass spectrometry to detect sphingolipids. Each lipid amount (in pmol) was normalized by the PI abundance (pmol/PI). **, *P* < 0.01, ***, *P* < 0.001, and ****, *P* < 0.0001 when groups were compared using one-way ANOVA with Dunnet’s posttest. Download FIG S8, TIF file, 2.1 MB. Copyright © 2019 Liu et al. 2019 Liu et al. This content is distributed under the terms of the Creative Commons Attribution 4.0 International license.

To verify this hypothesis, we first analyzed the levels of sphingolipids in the Δ*Molag1* mutant with LC-MS. Compared to its accumulation in the wild-type Guy11, the accumulation of the ceramide precursor sphinganine showed an increase in the Δ*Molag1* mutant (Fig. 7b), while the contents of C_18_-dihydroceramide, C_18_-ceramide, and C_18_-OH-ceramide decreased significantly. Correspondingly, we observed lower levels of C_19:2_/C_18_ GlcCers in the Δ*Molag1* mutant (Fig. 7b). Unexpectedly, although the levels of phytosphingosine, phytoceramide, and C_26_-dihydroceramide in the wild type and the Δ*Molag1* mutant did not change significantly, the mutant showed a marked depletion of IPC (18:0;3/26:0;0) (Fig. 7b; Fig. S8c). Overall, MoLag1 mainly catalyzes the production of C_18_-Cers, which are responsible for the synthesis of GlcCers.

**FIG 8:**
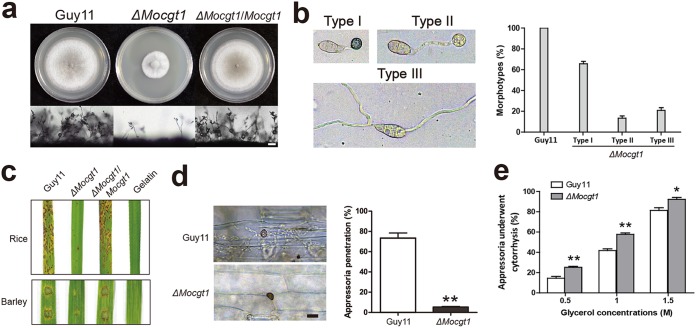
Biological functions of *MoCGT1*. (a) Colony growth and conidiophores of the indicated *M. oryzae* strains (scale bar = 20 μm). The Δ*Mocgt1* mutant showed reduced mycelial growth and conidiation. Data from inferential statistical tests of the conidiation assays are presented in Table S1j. (b) Appressorial morphotypes of Δ*Mocgt1* mutant and proportions of the three morphotypes (scale bar = 10 μm). (c) Attenuated virulence of the Δ*Mocgt1* mutant compared to Guy11. Detached rice or barley leaves were sprayed with conidial suspensions of *M. oryzae* strains and cultured for 4 to 7 days to allow disease development. (d) Decreased ability of appressorium to infect host in Δ*Mocgt1* mutant (scale bar = 10 μm). Infected barley leaves at 48 hpi were decolorized prior to microscopic observation of the infected cells in the epidermis. Data represent the mean values ± SD (*n* = 100, three independent experiments). Data were analyzed with the two-tailed *t* test comparison. Values marked with asterisks are statistically significant. **, *P* < 0.001 (df = 4, *P* = 0.000). (e) Percentages of appressoria (wild type and Δ*Mocgt1* strains) undergoing cytorrhysis under different concentrations of glycerol. Data represent the mean values ± SD (*n* = 100, three independent experiments). Data were analyzed with two-tailed *t* test comparison. Values marked with asterisks are statistically significant. *, *P* < 0.05; **, *P* < 0.001 (df = 4, *P* = 0.000).

We further identified the ortholog of the yeast gene encoding ceramide galactosyltransferase in M. oryzae (*MoCGT1*; MGG_10668). A previous study has shown that MoCgt1 is responsible for the biosynthesis of GlcCers (38). We obtained a null mutant of *MoCGT1* and were surprised to find that the colony morphology of the Δ*Mocgt1* mutant was similar to that of the Δ*Molag1* mutant. The Δ*Mocgt1* colonies showed slow growth and sparse mycelia (Fig. 8a). The Δ*Mocgt1* mutant also showed reduced conidiation, with the total number of spores being about 1% of that of the wild type (Fig. 8a; Table S1j). More importantly, similar to the results for myriocin treatment, the appressoria formed by the Δ*Mocgt1* mutant also displayed three different morphotypes. Type I was similar to that of Guy11, type II was immature and lacked the melanized appressorial cell wall, and in type III, the polarized growth of the conidial germ tube was perturbed, leading to a loss of appressoria (Fig. 8b). The pathogenicity of the Δ*Mocgt1* mutant on barley and rice leaves was also much lower than that of the wild type (Fig. 8c). Observations on barley leaves infected by conidia of Guy11 and the Δ*Mocgt1* mutant for 48 h showed that, while ∼74% of the wild-type appressoria successfully infected host leaves, only ∼6% of the appressoria of the Δ*Mocgt1* mutant could produce penetration pegs and colonize plants (Fig. 8d). In contrast to that of the wild-type Guy11, the appressorial turgor pressure of the Δ*Mocgt1* mutant was significantly decreased (Fig. 8e). The growth, sporulation, appressorium morphology, and pathogenicity of transformants were restored by reintroducing the wild-type *MoCGT1* gene (Fig. 8).

We also studied the role of IPC in appressorium formation by applying aureobasidin A (AbA), a specific inhibitor of inositol phosphorylceramide synthase (Ipc1), in the fungus (39). Both *in vitro* enzyme activity analysis and mycelial growth inhibition assays were conducted, and the results demonstrated that AbA can effectively inhibit the activity of Ipc1 in a dose-dependent manner in *M. oryzae* (Fig. S9a and b). Then, we studied the effect of AbA on appressorium development and found that the morphology and function of appressoria were not affected by AbA in the concentration range studied (Fig. S9c).

10.1128/mBio.01467-19.9 FIG S9 AbA had no effect on the morphology and function of appressoria of *M. oryzae*. (a) Inhibitory effects of AbA on mycelial growth of *M. oryzae*. The strains were cultured in the dark in complete medium (CM) with different concentrations of inhibitors. The colony diameters were measured after 3 days, and the inhibition rates were calculated. (b) *In vitro* analysis of Ipc1 activity in *M. oryzae*. Proteins from the cell lysates were treated with different concentrations of AbA for 5 minutes, and the Ipc1 activity was determined as conversion of NBD-C_6_-ceramide into NBD-C_6_-IPC by LC analysis. (c) The effect of AbA on appressorium. Conidia were treated with different doses of AbA and incubated on a hydrophobic surface for 24 h to observe the morphologies of appressoria. Conidia treated with different doses of AbA were inoculated onto barley leaves for 4 days to observe the pathogenicities of appressoria (scale bar = 10 μm). Download FIG S9, TIF file, 2.2 MB. Copyright © 2019 Liu et al. 2019 Liu et al. This content is distributed under the terms of the Creative Commons Attribution 4.0 International license.

**FIG 9:**
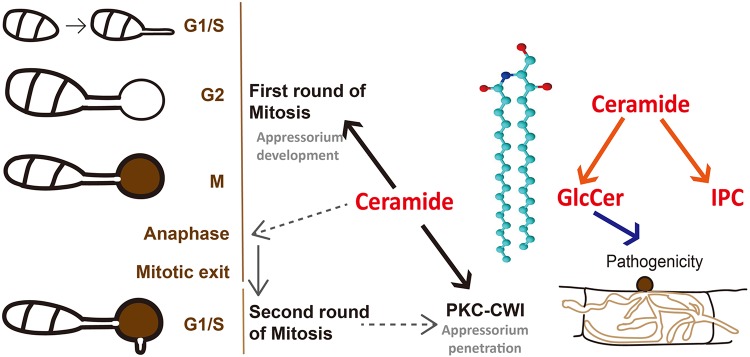
Model for the role of ceramide in appressoria of *M. oryzae*. Red font shows metabolites, and orange arrows represent metabolic processes. Black and gray arrows represent the molecular mechanisms of metabolites involved in appressoria. Ceramide controls the first round of mitosis during normal appressorial maturation. Furthermore, ceramide was also found to act upstream from the PKC-CWI pathway during appressorium repolarization (black arrows). Based on the published results on rice blast fungus, we speculate that ceramide first affects the anaphase stage of the cell cycle, thus preventing the occurrence of the second S phase of the cell cycle; the absence of S phase affects the transmission of the PKC-CWI pathway, resulting in a defect in penetration peg formation and the loss of pathogenicity in appressoria (gray arrows). On the other hand, ceramide can be used as a substrate to participate in the synthesis of GlcCer and IPC, and our research found that only GlcCer is directly related to the pathogenic process of rice blast fungus (blue arrow).

Combined with the above-described results, we conclude that the complex sphingolipid GlcCer is involved in the pathogenesis of the rice blast fungus and the loss of pathogenicity of the Δ*Molag1* mutant is due to the loss of GlcCer production therein (Fig. 9).

## DISCUSSION

Our study offers an illustrative example of how untargeted metabolomics analyses can lead to biological insights with important practical implications. The fact that ceramides function in fungal pathogenesis against plant hosts seems plausible in light of the findings on sphingolipids in animals. It was previously assumed that ceramides and other sphingolipids present in biological membranes function as purely supporting structural elements. However, recent studies have established that ceramide (that is, a sphingolipid lacking further modification) can participate in a variety of signaling processes, including regulation of cellular differentiation and proliferation and as an initiator of programmed cell death (40, 41). In M. oryzae, previous studies have shown that treatment of fungi with various ceramide compounds specifically triggered the initiation of appressorial development, thus suggesting that ceramides could somehow be perceived by or be involved in such signal transduction pathways in *M. oryzae* (42). Our study showed that the absence of ceramide elevated the sensitivity to cell wall stress in *M. oryzae.* We deduced that cell wall integrity was certainly disrupted due to the structural defects caused by reduction in or loss of sphingolipids. Meanwhile, the results on the phosphorylation status of Mps1 and the involvement of ceramide signaling in modulating the PKC-based CWI pathway support this hypothesis/working model. From CWI to PKC signaling, this process has a cascading effect (43). Impaired sphingolipid signaling resulted directly in defects in membrane integrity, and the PKC pathway was thereby impaired/impacted in an indirect manner. The above-mentioned results are also consistent with studies in yeast (43, 44). In addition to the role of resisting cell wall stress, the CWI pathway, involving the Mps1 MAPK, is also known to be necessary for penetration peg formation (17, 34). Notably, the wild-type strain Guy11 appressoria treated with myriocin/FB1 and the appressorium-like structures produced by the Δ*Molag1* strain showed the absence of penetration pegs. Besides, the Δ*mps1* mutant showed reduced conidiation, indicating that Mps1 signaling also regulates the conidiation in *M. oryzae* (17). The phenotypes of the Δ*Molag1* mutant support the fact that Mps1 signaling is required for conidiation and appressorium function in *M. oryzae* and explain why sphingolipid biosynthesis influences pathogenicity in the rice blast fungus. Furthermore, the inhibitor-mediated disruption of ceramide biosynthesis altered glycogen metabolism and the trafficking/stability of lipid droplets, the two major energy sources that facilitate appressorial development. Thus, the vital role of ceramide is in the regulation not only of signal transduction but also metabolism processes in the rice blast fungus.

It was also highly conspicuous that our metabolomics analysis showed that the increasing and subsequently decreasing trend in ceramide levels was highly similar to that observed for the turgor pressure-generating compatible-solute glycerol. A similar trend was also found for phosphatidylethanolamine (PE), to which the autophagy protein Atg8 conjugates to initiate autophagosome biogenesis (45). Normal appressorial development is known to require mitosis and autophagic cell death (6). G_1_/S and G_2_/M cell cycle checkpoints mediate the maturation of the appressorium, after which the three remaining nuclei in the conidium are degraded via autophagy (10, 11). In *M. oryzae*, blocking the later stages (anaphase and mitotic exit) of the cell cycle causes a range of nuclear distribution defects, including a failure in nuclear migration to the developing appressorium (10). Furthermore, arresting cells in a preanaphase state also causes different degrees of maturation of the appressoria (10, 11). We therefore reasoned that early sphingolipids affect the anaphase stage of mitosis during appressorium development and the degradation of nuclei in conidia. The influence of different stages of the cell cycle on the formation of penetration pegs has also been studied (11). It is now known that the second round of S-phase progression, but not the later stages of the cell cycle, affect the formation of penetration pegs. Our observation that treatment of developing appressoria with myriocin affected the appressorial maturation and subsequent penetration peg formation thus suggests a potential cellular mechanism(s): disruption of early sphingolipid biosynthesis disturbs the later stage(s) of the first round of mitosis (anaphase), thereby preventing the occurrence of consequent mitotic exit and the second round of S phase. We also speculate that the second round of S phase regulates the downstream PKC signaling pathway, thus modulating the formation of penetration pegs in *M. oryzae* (Fig. 9).

Besides their roles in signaling, ceramides are precursors of the complex sphingolipids GlcCer and IPC. GlcCers have been shown to be important for pathogenesis in fungal phytopathogens. For example, the inhibitory binding of fungal sphingolipid-derived glucosides by a specific antibody reduced disease symptoms on tomato leaves inoculated with Botrytis cinerea (46). The virulence of Fusarium graminearum GlcCer mutants is reduced on wheat (47). When sprayed on rice leaves, GlcCers elicit phytoalexin production, induce resistance proteins, and promote plant resistance responses against subsequent infections (48). Another complex sphingolipid, IPC, is a fungus-specific metabolite that is essential for the growth and survival of fungi. In S. cerevisiae, when the sphingolipid synthesis pathway is blocked, IPC is no longer synthesized and yeast dies rapidly (49). Studies have also shown that IPC is involved in the pathogenesis of some other fungi. In Cryptococcus neoformans, downregulation of IPC1 markedly lowers the expression of certain virulence traits and impairs its pathogenicity (50). Furthermore, treatment of B. cinerea with AbA prevents its infection in tomato fruits (51). Here, we demonstrate that ceramides produced by MoLag1 function in fungal pathogenesis. Furthermore, the analysis of sphingolipid levels in the Δ*Molag1* mutant indicates that MoLag1 mainly synthesizes ceramides for GlcCers. Finally, our results prove that GlcCer plays an important role in the pathogenic process of the rice blast fungus. We also used AbA to inhibit the activity of Ipc1 and found that IPC is mainly involved in the growth process of *M. oryzae*, rather than the pathogenic process.

Collectively, our results reveal a wide range of metabolites that might take part in appressorium development. We also further deepen our understanding of ceramide in the morphology and pathogenicity of appressoria in *M. oryzae*. We anticipate that this new metabolic information will help to broaden the scope of future analysis of appressorium morphogenesis and function, and additional functional characterization of ceramides would also facilitate the understanding of the blast fungus pathogenesis process. Extending these metabolic insights into a practical application, we were here able to successfully block the pathogenicity of *M. oryzae* in rice and barley by chemically disrupting two different sphingolipid-producing enzymes. Thus, the sphingolipid biosynthesis pathway can reasonably be viewed as an important/critical target that can be exploited for the identification and development of novel disease control agents.

## MATERIALS AND METHODS

### Fungal culture conditions and appressorium sampling.

The wild-type strain of *M. oryzae* (Guy11) and its mutants were cultured and maintained on complete medium (CM) plates at 25°C with a 16-h-light and 8-h-dark cycle as described previously (29). For cultivation in the liquid medium, mycelia from a 6-day-old colony were transferred to liquid CM for 3 days with shaking (150 rpm) at room temperature. For incubating appressoria, conidia harvested from a 10-day-old colony were resuspended in sterile distilled water and incubated on the hydrophobic surface of projection transparency film (Terylene resin; Gaoke, China) at 25°C. Appressoria were harvested in chilled sterile water at the chosen time points (0 h, 6 h, 12 h, 18 h, 24 h, 36 h, and 48 h) and quickly stored at –80°C prior to lyophilization. Each group consisted of 6 replicates. Two schemes for the collection of appressoria were compared. In method 1, the quantified conidia (5 × 10^5^ conidia/ml, 30 ml) were germinated and collected to extract the metabolites; this is the most common method used for extracting RNA and proteins from appressoria (52). In method 2, the induced appressoria were collected without determining their concentration (conidia/ml), and the metabolites were extracted from 10 mg of freeze-dried samples. We collected samples at two phases of germinating conidia (0 h and 24 h) using both methods and analyzed the extracted metabolites by GC-MS. The data were subjected to PCA to compare the different methods (Fig. S1a and b in the supplemental material). It was found that the reliability of method 2 data is better than that of method 1 data. Method 2 was thereby chosen for the experiments reported in this study.

### Untargeted metabolomic profiling. (i) GC-MS sample preparation.

The freeze-dried samples were ground to a uniform powder and stored at –80°C prior to metabolomics analyses. The powder was soaked in 1.5 ml of an extraction solvent containing isopropanol/acetonitrile/water (3:3:2, by volume) with 15 μl (2 mg/ml) of tridecanoic acid as an internal standard. All extracts were sonicated for 1 h and centrifuged for 10 min at 14,000 rpm and 4°C. Supernatants were dried under a nitrogen flow on an N-Evap nitrogen evaporator (Organomation, USA). The extract was then oximated with 100 μl methoxyamine hydrochloride for 90 min at 37°C and silylated with 100 μl methyl-trimethyl-silyl-trifluoroacetamide (MSTFA) for 60 min at 60°C in order to increase the volatility of the metabolites.

**(ii) GC-MS analysis.** The metabolite analysis was carried out on an Agilent 7890A series gas chromatograph (GC) system using a 5975C mass selective detector (MSD) equipped with an Agilent G4513A injector (Agilent, USA). An Agilent DB-5MS column (0.25 μm, 0.25 mm by 30 m) was used with the column temperature set at 70°C for the first 4 min and then increased by 15°C/min to 310°C over 15 min. The injection temperature was set as 300°C, and the injection volume was 1 μl with a 10:1 split ratio. Helium (99.9995%; Jingong, China) was applied as a carrier gas, and the column flow rate was 1.2 ml/min under the control of a linear velocity control model. Prior to the instrumental analysis, the mass spectrometer was tuned using perfluorotributylamine (PFTBA) to obtain optimum performance. The mass spectrum scanning scope was set to 33 to 600 *m/z* in the full scan mode. The scan speed was 2.59 scans/s, and the solvent hold-up time was 5.0 min. The temperatures of the interface and the ion source were adjusted to 280 and 230°C, respectively. The detector voltage was maintained at 1.2 kV, and the electron impact (EI) model was selected to achieve ionization of the metabolites at 70 eV.

**(iii) LC-MS sample preparation.** The powder of freeze-dried tissue samples was soaked in an extraction solvent containing isopropanol/acetonitrile/water (3:3:2, by vol), with 10 μg/ml umbelliferone used as an internal standard. All extracts were sonicated for an hour and centrifuged for 10 min (14,000 rpm at 4°C). Supernatants were collected for LC-MS analysis.

**(iv) LC-MS analysis.** LC-MS analysis was performed on an Agilent 1290 liquid chromatograph (LC) system coupled to an Agilent 6540 ultrahigh definition (UHD) quadrupole time of flight (Q-TOF) mass spectrometer equipped with an electrospray ionization source. An Agilent SB-C_18_ column (rapid resolution high definition [RRHD], 1.8 μm, 2.1 by 100 mm) was used. The samples were analyzed in positive ion mode. Mobile phases A and B were 95% water/5% acetonitrile with 0.1% formic acid and 5% water/95% acetonitrile with 0.1% formic acid, respectively, and the gradient was as follows: *t* = 2 min, 1% mobile phase B; *t* = 3 min, 35% B; *t* = 8 min, 60% B; *t* = 10 min, 70% B; *t* = 20 min, 83% B; *t* = 22 min, 90% B; *t* = 26 min, 100% B; *t* = 28 min, 100% B. The flow rate was 0.3 ml/min. Five-microliter aliquots of the sample were loaded for each individual analysis. The capillary voltage and spray shield were set to 3,500 V. The sheath gas was set to 10 liters/min at a temperature of 350°C. The nebulizer gas was set to 12 liters/min at 350°C. Spectra were acquired over a range of *m/z* 50 to 1,200. The collision energy in the MS/MS mode was set to 20 V. LC-MS-grade acetonitrile, isopropanol, distilled water, and formic acid (HCOOH) were obtained from Fisher Scientific, UK. All standards used in this study were purchased from Sigma-Aldrich, USA.

### Analysis of fungal sphingolipids by LC-MS.

The procedures for sphingolipid extraction and mass spectrometry analysis were performed as described before (53). Briefly, Guy11 strains were cultured in liquid CM for 2 days and then collected and ground with liquid nitrogen. Lipids were extracted by Mandala extraction (54), Bligh and Dyer extraction (55), and base hydrolysis (56) successively. The mixed extracts and internal standards were dried and redissolved in mobile phase B. MS analysis was performed on a Thermo Accela high-performance liquid chromatography (HPLC) system coupled to a Thermo TSQ Quantum Ultra mass spectrometer (Thermo, USA).

### *In vitro* activity of Ipc1.

The Ipc1 activity assays were carried out by following previously described methods (50, 57), with slight modifications. Briefly, Guy11 strains were cultured in liquid CM for 2 days and then collected and ground with liquid nitrogen. The fractured tissues were resuspended in lysis buffer (50) and centrifuged at 2,500 × *g* for 10 min at 4°C. Proteins in the supernatant were quantified by the Bradford method (58). Proteins (150 μg) were preincubated with different concentrations of aureobasidin A (AbA) for 5 min and then incubated at 30°C for 30 min in assay buffer (57) containing 0.1 mM C_6_-NBD-ceramide (N-{6-[(7-nitro-2-1,3-benzoxadiazol-4-yl)amino]hexanoyl}-d-erythro-sphingosine; ApexBio, USA) and 2 mM phosphatidylinositol (PI; Sigma-Aldrich, USA) in a final reaction mixture volume of 100 μl. The reaction was quenched with 500 μl 0.1 M HCl in methanol. One milliliter chloroform and 1.5 ml MgCl_2_ (1 M) were added into the reaction mixture, and the phases were separated by centrifugation at 1,000 × *g* for 10 min. The bottom chloroform phases were extracted, and the fluorescent substrate and product (NBD-ceramide or NBD-IPC, N-{6-[(7-nitro-2-1,3-benzoxadiazol-4-yl)amino]hexanoyl}-inositol-phosphatidylceramide) were analyzed using a Waters 600 HPLC coupled with a 2475 fluorescence detector (Waters, USA). An Atlantis T3 (5 μm, 4.6 by 250 mm; Waters, USA) column was used with a gradient of 50% mobile phase A (0.1% CH_3_COOH)–50% mobile phase B (CH_3_CN) to 10% A–90% B; the maximum excitation and emission wavelengths were set at 465 nm and 530 nm, respectively.

### Data analysis.

Metabolomics data were initially converted into the mzXML data format and sequentially handled by XCMS software running under R environment version 2.3.1 for metabolic feature detection and chromatographic matching. For GC-MS analysis, the NIST 14.0 standard mass spectral database was used to identify metabolites (in the GC-MS chromatograms). Metabolites were identified based on their retention index and mass spectral similarity (more than 70%) to the database; some of these structural identifications were validated using known standard compounds. For LC-MS analysis, each *m/z* value and MS/MS spectrum of the ionization product were matched in the METLIN database (http://metlin.scripps.edu/), HMDB database (http://www.hmdb.ca/), and Lipid Maps database (http://www.lipidmaps.org/) with parameters of ppm = 30, adducts = [M+H]^+^, and positive ion mode. For sphingolipids and NBD-ceramide/NBD-IPC analysis, data were extracted using Thermo Xcalibur 2.2 software and Waters Empower software, respectively. Principal-component analysis (PCA) and orthogonal partial least-squares discriminant analysis (OPLS-DA) were used in the data analysis implemented in SIMCA-P version 14.1 (Umetrics AB, Sweden). The significance was expressed by using the variable importance in projection (VIP) values of the OPLS-DA SIMCA-P analysis and using one-way analysis of variance (ANOVA) or two-tailed *t* test conducted in Statistical Product and Service Solutions software (IBM, USA). VIP values exceeding 1.0 and Student’s *t* test *P* values of less than 0.05 were considered to show a significant difference. Differentially accumulated metabolites were analyzed using the KEGG database (http://www.kegg.jp/) for pathway analysis.

### Chemical inhibition and complementation.

Conidia treated with myriocin (LKT Labs, USA), FB1 (ApexBio, USA), rapamycin (ApexBio, USA), cAMP (Sigma-Aldrich, USA), Go 6983 (Sigma-Aldrich, USA), and aureobasidin A (Solarbio, China) were drop inoculated onto the hydrophobic membrane for 24 h to observe the formation of appressoria. For chelerythrine chloride (ApexBio, USA) treatment assays, inhibitors were added to conidial suspensions at 4 h following incubation, a time when almost all of the conidia had germinated.

Glycogen and lipid bodies were observed during development at 0 h, 8 h, 16 h, and 24 h of incubation with KI/I_2_ and Nile red staining as previously described (20). Fluorescent microscopy observations were carried out using an Eclipse 80i microscope (Nikon, Japan). In order to visualize nuclear degeneration in *M*. *oryzae*, Guy11 expressing histone H2B:GFP treated with 100 μM myriocin was examined by epifluorescence microscopy at 24 h. Mycelia of the Δ*Molag1* strain grown with shaking in liquid CM with C_2_-ceramide (Sigma-Aldrich, USA) were inoculated onto the surface of detached barley leaves for pathogenesis analysis.

### Targeted gene disruption, complementation, and phenotypic analysis.

*MoLAG1* was identified through a BLASTp search of the *M. oryzae* genome database (http://www.broadinstitute.org/annotation/genome/magnaporthe_grisea/MultiHome.html) against the yeast LAG1 protein. Targeted gene disruption was carried out using a previously described high-throughput gene knockout system (59). The targeted gene deletion vector was introduced into the wild-type strain Guy11 via an Agrobacterium tumefaciens-mediated transformation (ATMT) method. For complementation, the full-length genomic gene sequence with its native promoter sequence was cloned from *M. oryzae* genomic DNA and subcloned into the vector pKD5. The primers used in this study are presented in Table S1g. For vegetative growth, development of conidia on conidiophores, conidiation, incipient cytorrhysis assays, pathogenicity tests on barley (Hordeum vulgare) and rice CO-39 (Oryza sativa), and penetration assays were performed as described previously (29, 59).

### Western blot analysis.

Vegetative hyphae used for protein extraction were harvested 72 h after culture in liquid CM. The protein extraction assays were performed as described previously (60). Total proteins were separated on SDS-PAGE gels and transferred to polyvinylidene difluoride membrane for Western blot analysis. Expression and phosphorylation of Mps1 were detected by using PhosphoPlus p44/42 MAP kinase antibody kits (Cell Signaling Technology, USA).
