# Supplementary figures and images for: Metabolomics Analysis Identifies Sphingolipids as Key Signaling Moieties in Appressorium Morphogenesis and Function in Magnaporthe oryzae
Source: mBio. 2019 Aug 20;10(4):e01467-19. doi: 10.1128/mBio.01467-19 (PMC6703424; doi:10.1128/mBio.01467-19)

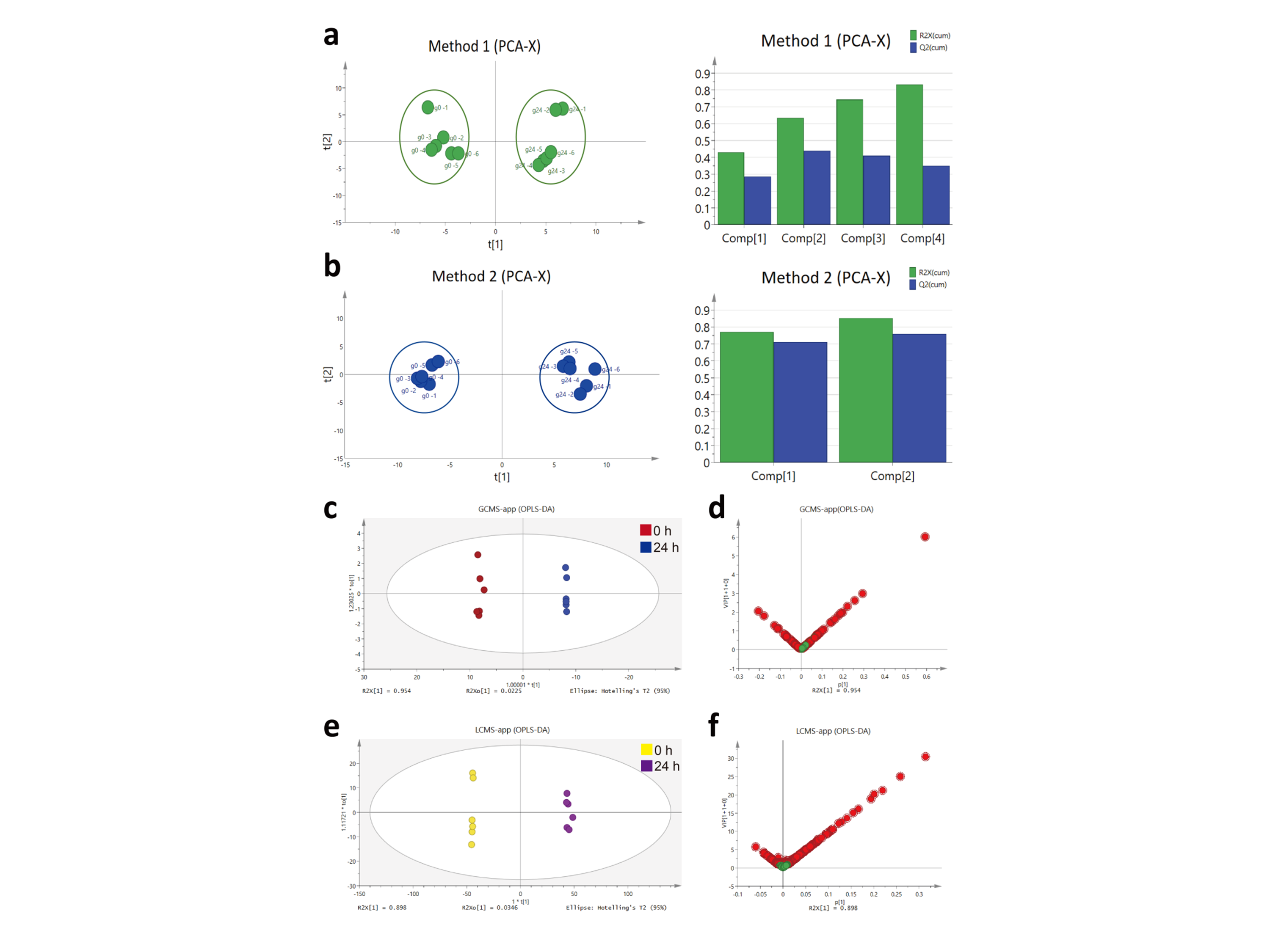

Supplement: FIG S1 [file mBio.01467-19-sf001.tif]

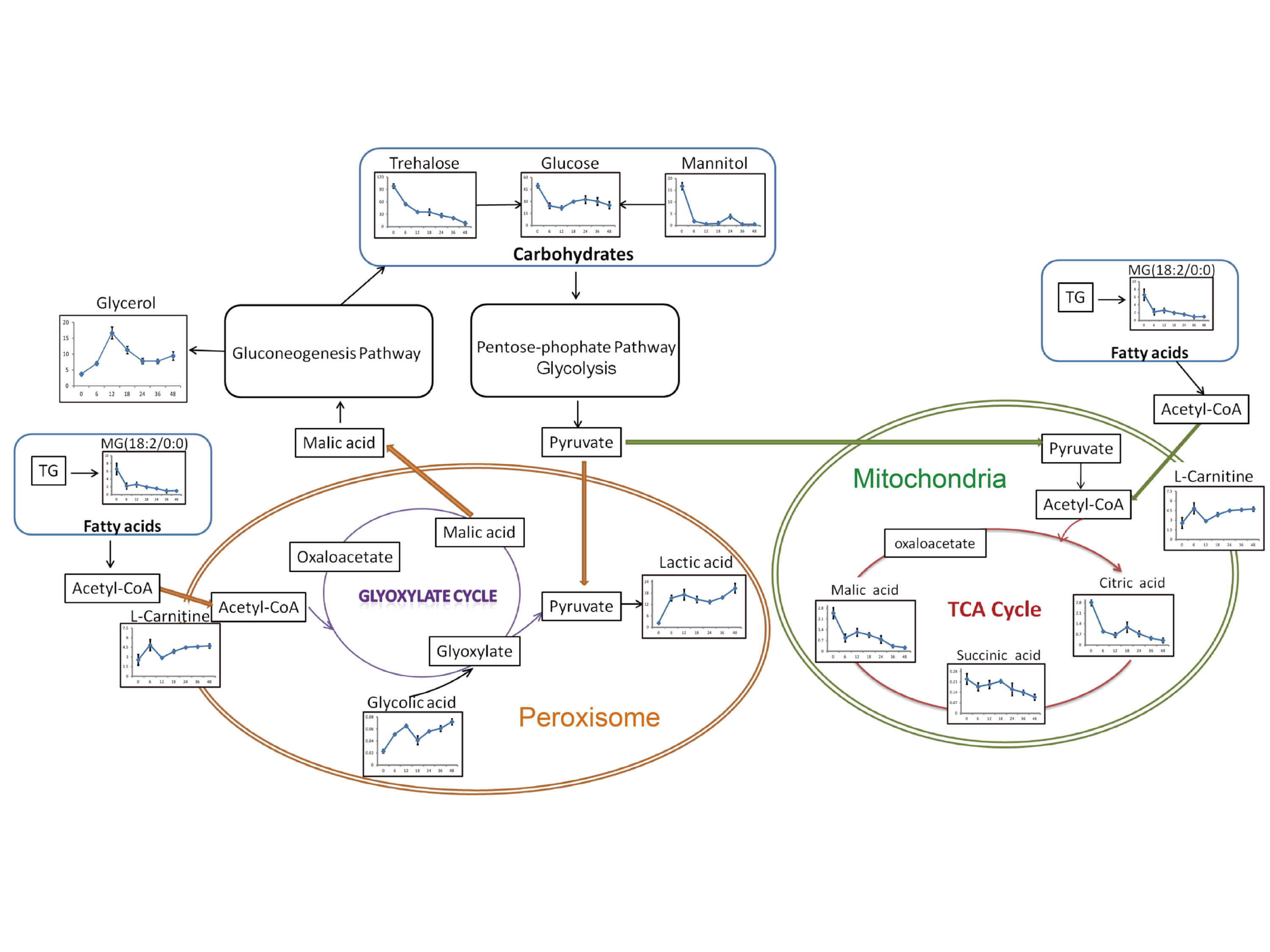

Supplement: FIG S2 [file mBio.01467-19-sf002.tif]

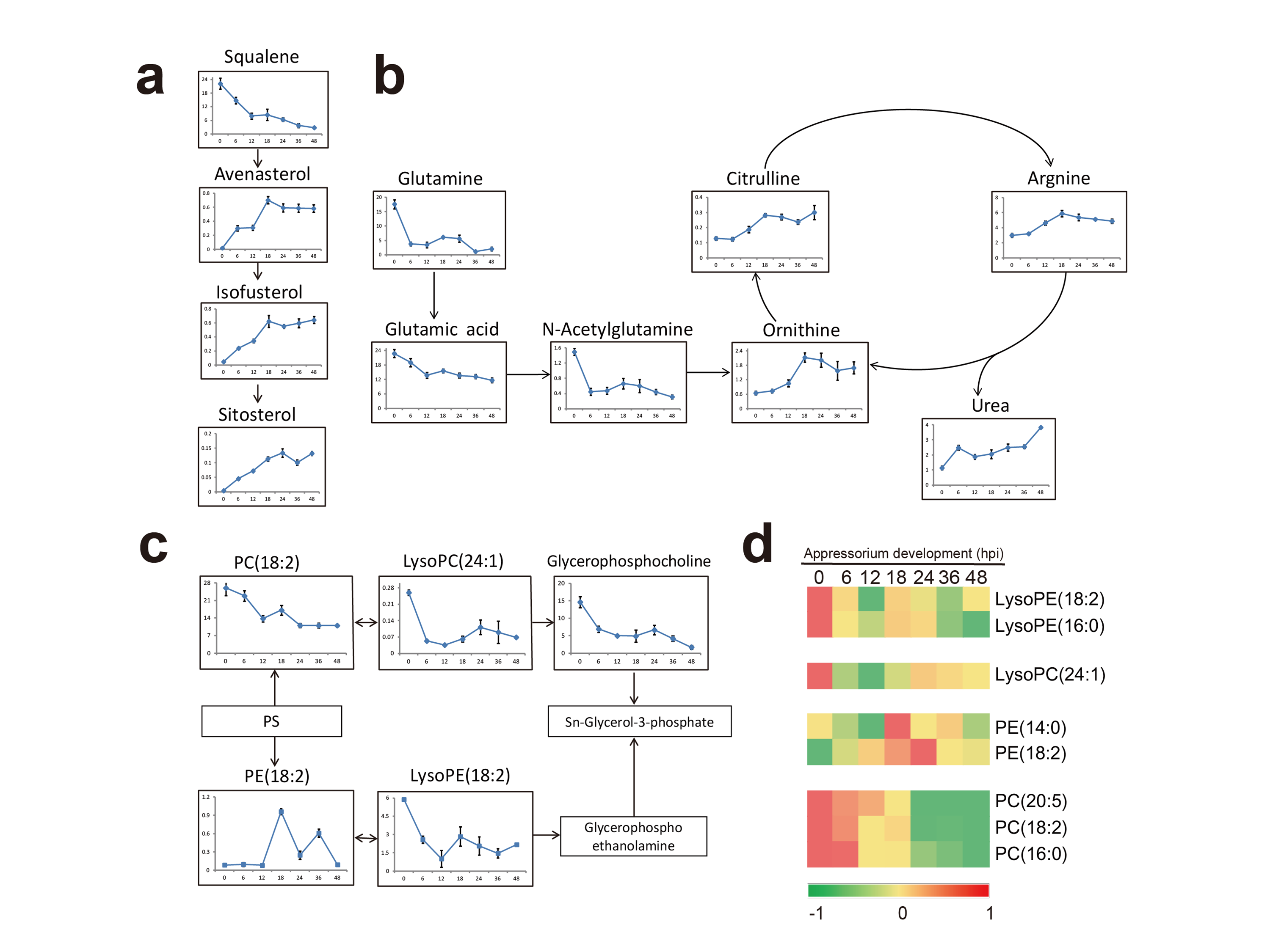

Supplement: FIG S3 [file mBio.01467-19-sf003.tif]

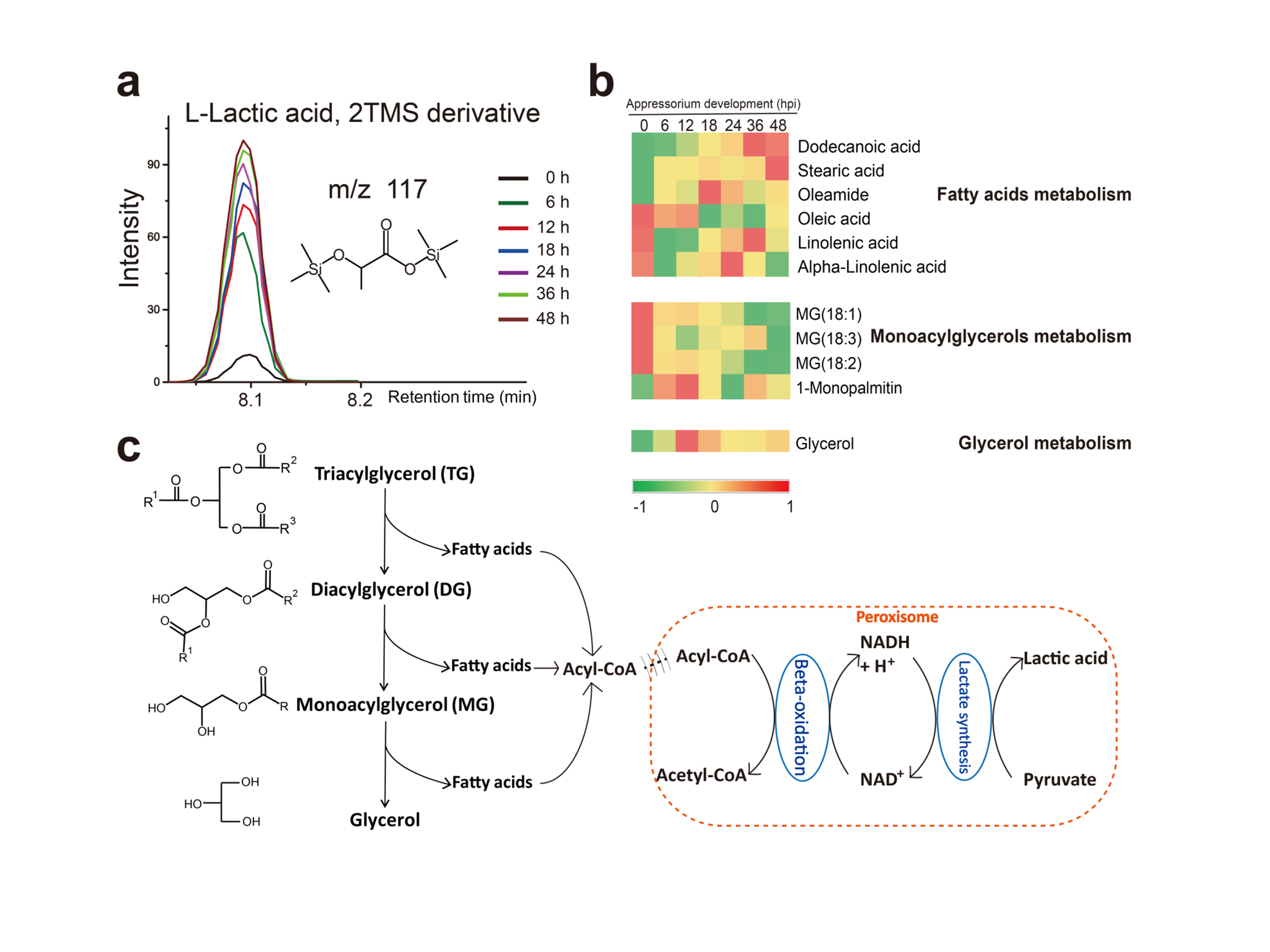

Supplement: FIG S4 [file mBio.01467-19-sf004.tif]

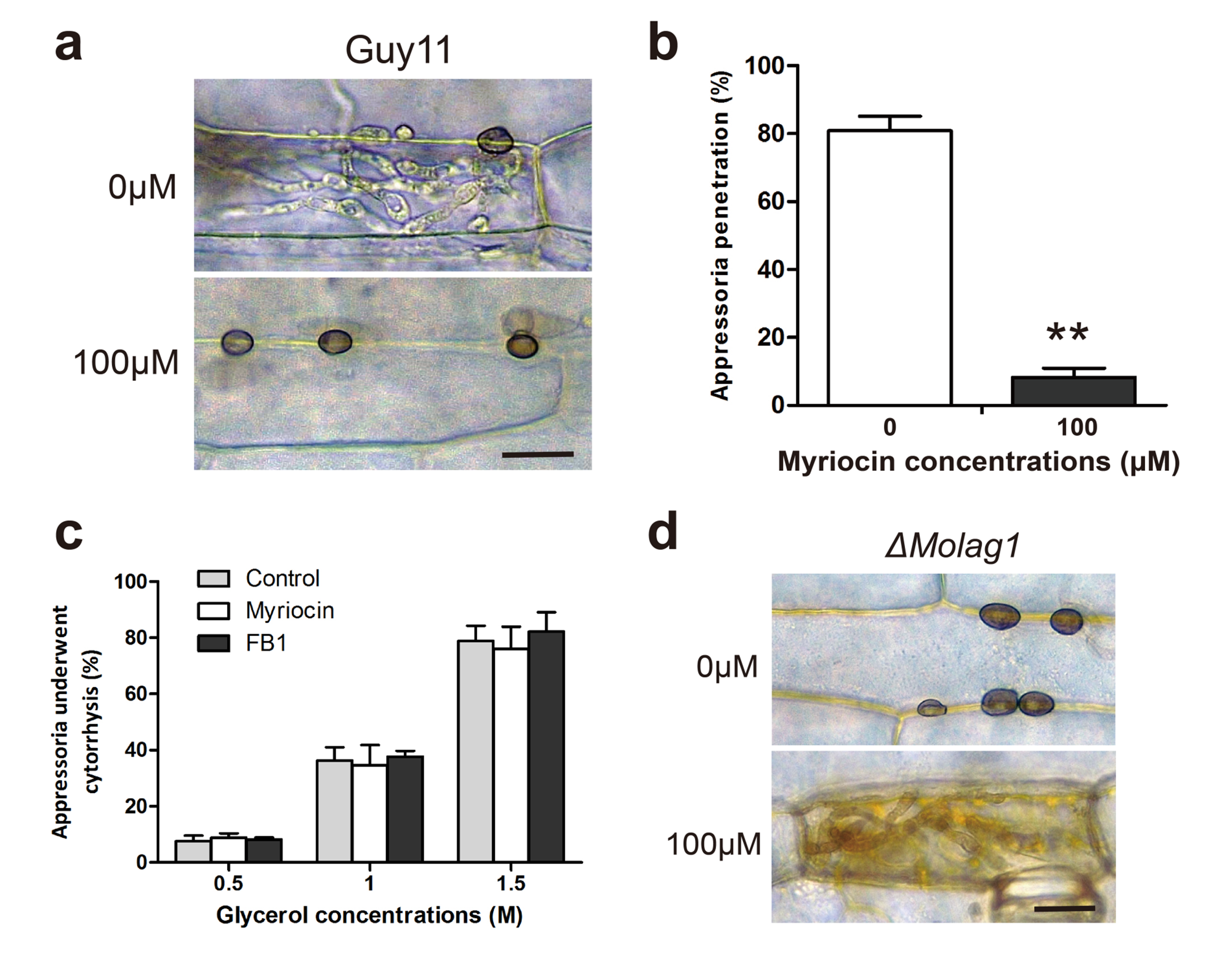

Supplement: FIG S5 [file mBio.01467-19-sf005.tif]

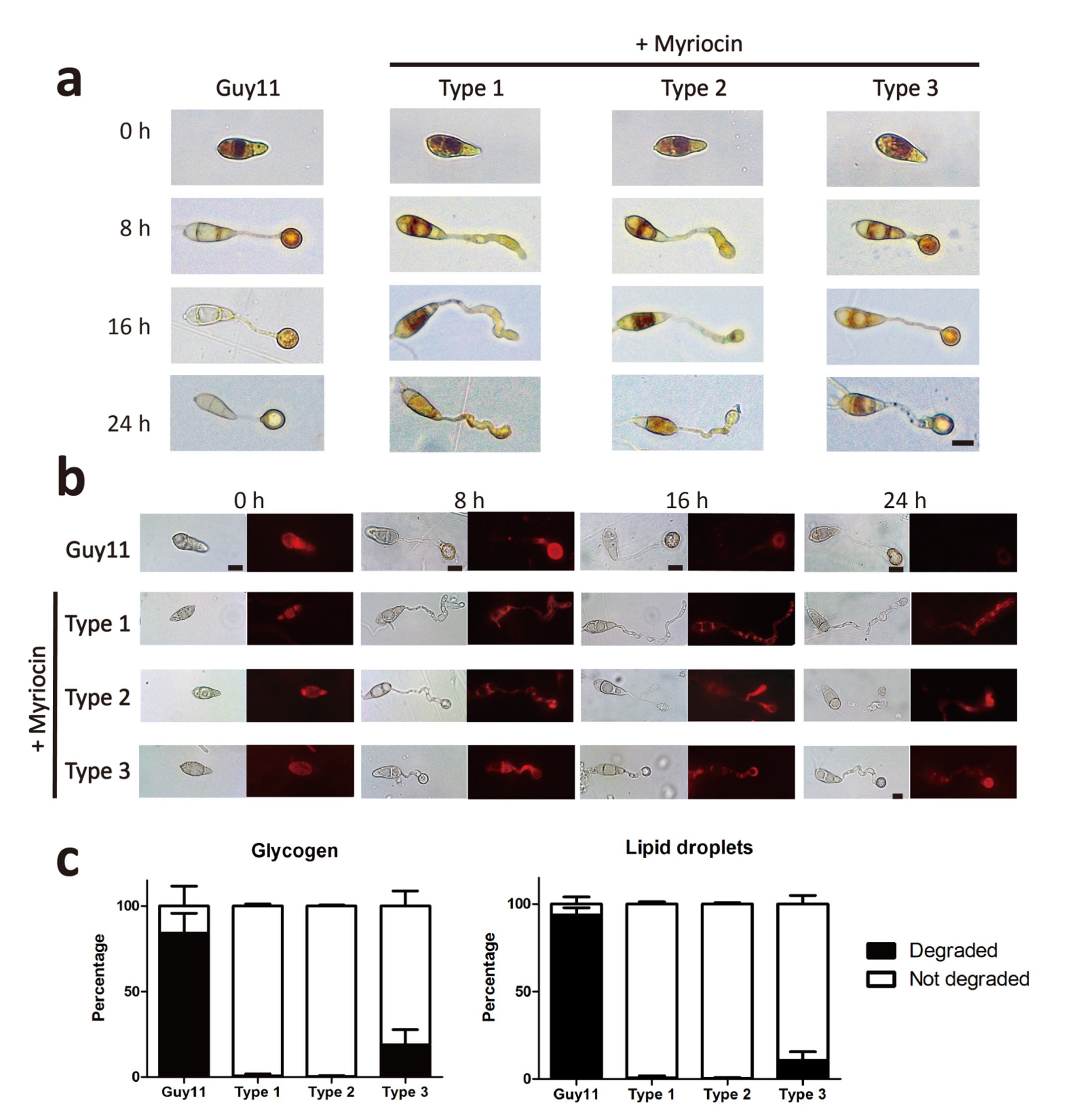

Supplement: FIG S6 [file mBio.01467-19-sf006.jpg]

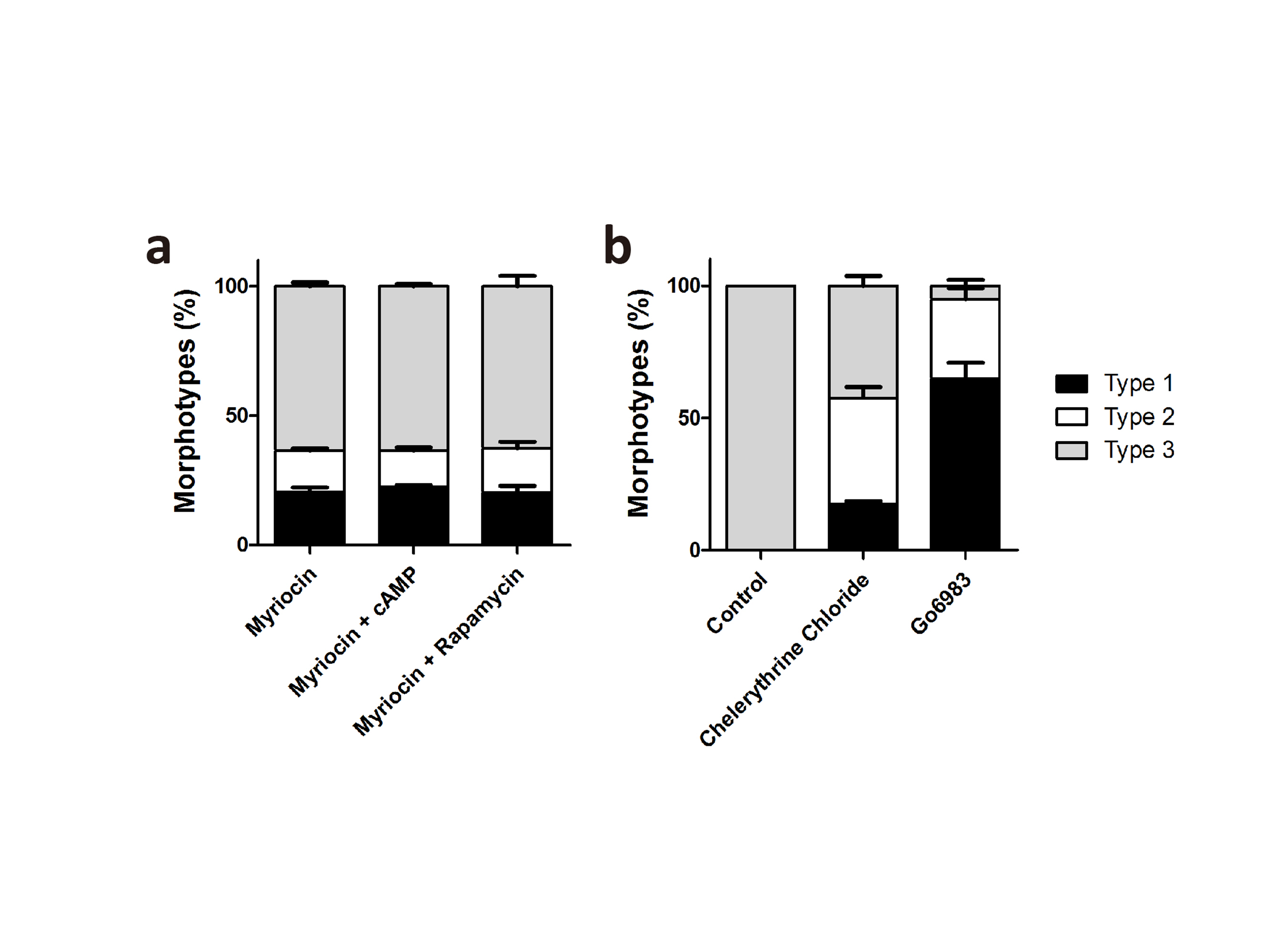

Supplement: FIG S7 [file mBio.01467-19-sf007.tif]

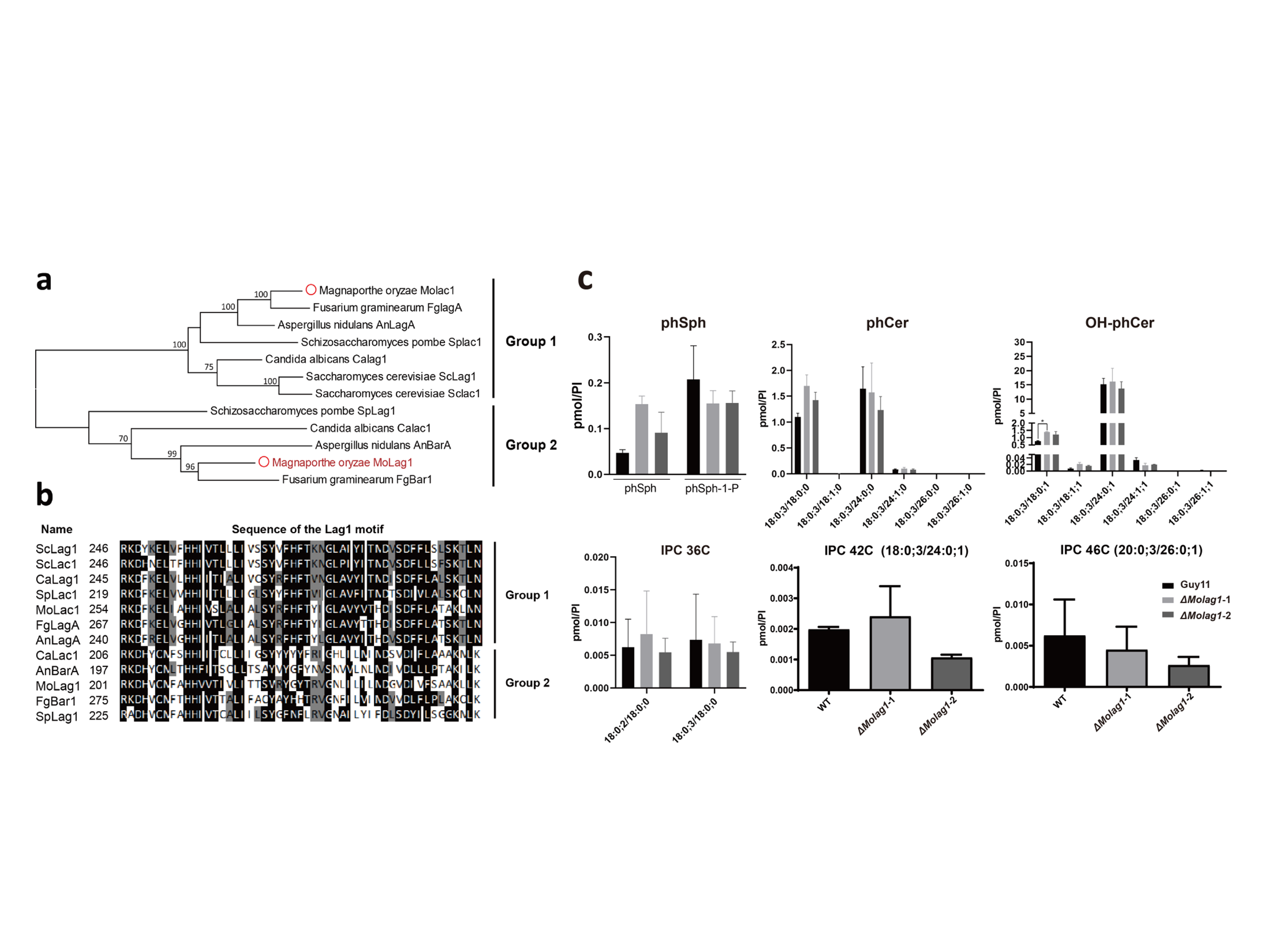

Supplement: FIG S8 [file mBio.01467-19-sf008.tif]

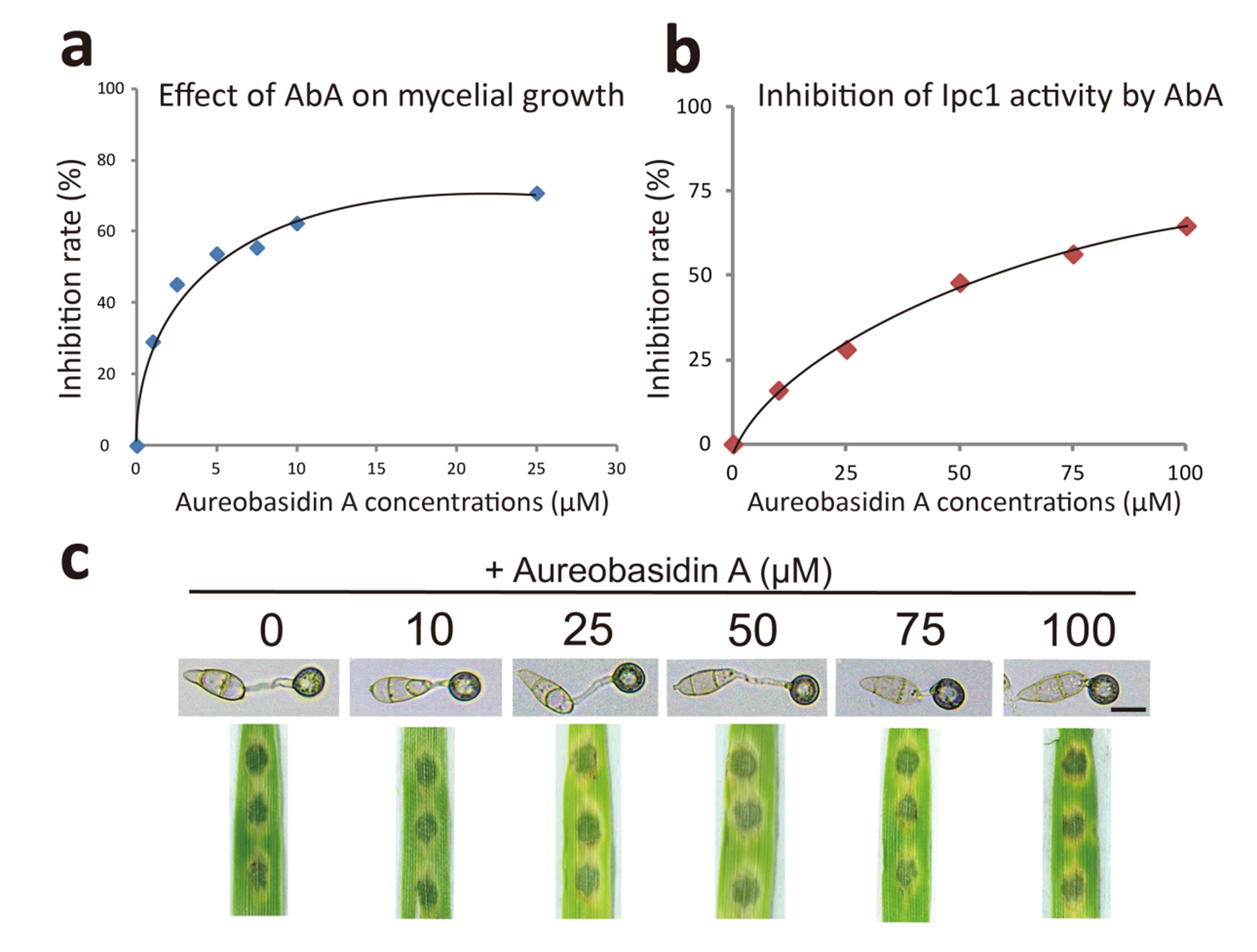

Supplement: FIG S9 [file mBio.01467-19-sf009.tif]
